# Diet, Physical Activity, Lifestyle Behaviors, and Prevalence of Childhood Obesity in Irish Children: The Cork Children’s Lifestyle Study Protocol

**DOI:** 10.2196/resprot.3140

**Published:** 2014-08-19

**Authors:** Eimear Keane, Patricia M Kearney, Ivan J Perry, Gemma M Browne, Janas M Harrington

**Affiliations:** ^1^Department of Epidemiology and Public HealthUniversity College CorkCorkIreland

**Keywords:** overweight, obesity, children, diet, physical activity, lifestyle, Ireland

## Abstract

**Background:**

Childhood obesity is complex, and its aetiology is known to be multifaceted. The contribution of lifestyle behaviors, including poor diet and physical inactivity, to obesity remains unclear. Due to the current high prevalence, childhood obesity is an urgent public health priority requiring current and reliable data to further understand its aetiology.

**Objective:**

The objective of this study is to explore the individual, family, and environmental factors associated with childhood overweight and obesity, with a specific focus on diet and physical activity. A secondary objective of the study is to determine the average salt intake and distribution of blood pressure in Irish children.

**Methods:**

A cross-sectional survey was conducted of children 8-11 years old in primary schools in Cork, Ireland. Urban schools were selected using a probability proportionate to size sampling strategy, and a complete sample of rural schools from one area in Cork County were invited to participate. Information collected included physical measurement data (anthropometric measurements, blood pressure), early morning spot and 24 hour urine samples, a 3 day estimated food diary, and 7 days of accelerometer data. Principal- (school head) reported, parent/guardian-reported, and child-reported questionnaires collected information on lifestyle behaviors and environmental attributes. The Cork Children’s Lifestyle Study (CCLaS) was designed by the Department of Epidemiology and Public Health in University College Cork, Ireland in 2011 and 2012. Piloting and modification of study methods was undertaken. Data collection took place between April 2012 and June 2013.

**Results:**

Overall, 27/46 schools and 1075/1641 children, of which 623 were boys, participated. Preliminary data analysis is underway. It is anticipated that the results of the CCLaS study will be available in late 2014.

**Conclusions:**

The CCLaS study has collected in-depth data on a wide range of individual, family, social, and environmental correlates which will allow us to access multilevel influences on childhood obesity. This study will contribute to the evidence base by highlighting current knowledge and gaps regarding the predominant drivers of childhood obesity.

## Introduction

### Childhood Obesity, Extent of the Problem

The worldwide prevalence of childhood obesity has increased significantly over the past three decades, particularly in North America and Western Europe [1,2]. Currently, over one in four Irish children are overweight or obese, and similar estimates are found in many other developed countries [1,3]. Childhood obesity can affect not only current health, but health in later life as well, and is a risk factor for metabolic syndrome, cardiovascular disease, and type 2 diabetes mellitus [4-7]. With the high prevalence and known adverse consequences of being obese [8,9], childhood obesity remains an urgent public health priority requiring current, detailed, and reliable data to further understand its aetiology, and to inform public health policies and interventions [10,11].

### Environmental and Lifestyle Factors

Obesity is a complex problem [12], and occurs as a result of a persistent positive energy balance where energy intake is greater than energy output [13]. Social ecological theory suggests that multiple levels of influence including individual, family, community, and organizational factors can enable or constrain health related behaviors that should to be considered when researching the determinants of obesity [14]. There is increasing consensus that environmental and lifestyle factors, rather than genetic or biological factors, are the primary drivers of the current childhood obesity epidemic [15-18]. A number of likely determinants of obesity have been identified including poor diet, physical inactivity, sedentary behavior, low socioeconomic status, and the built neighborhood environment [19-24].

There is a general perception that poor diet and physical inactivity are major contributors to the current obesity epidemic [25]. However, the relative contribution of poor diet and physical inactivity to childhood obesity are not well understood [26-29]. For example, little is known about dietary behaviors including food choice [30] and salt intake in children [31]. High salt intake is associated with poor diet [32,33], high blood pressure (BP) [34], and increased energy intake in children [35]. However, the association between childhood obesity and salt remains understudied, with some research indicating that salt may be indirectly associated with obesity through poor dietary choices including sugar sweetened beverage intake [35]. This is of concern as dietary behaviors [36] are established at an early age, and both obesity and BP track throughout one’s life [9,37].

The complex interplay between lifestyle patterns and environmental factors further complicates uncovering pathways to obesity [38]. Studies containing in-depth data on the association between a broad range of lifestyle factors and multiple measures of weight status are sparse, particularly in the Republic of Ireland. A small number of Irish studies have assessed diet, physical activity, or weight status in children, but most have only collected data on either physical activity or diet. In addition, most have used self-reported measures of weight status or physical activity, and little evidence is available on the wider environmental determinants of lifestyle patterns and obesity [39-41]. As the Cork Children’s Lifestyle Study (CCLaS) collected in-depth data on diet, physical activity, and weight status, this provides a unique opportunity to gain a deeper understanding on the multilevel influences associated with childhood obesity in Ireland.

The CCLaS study aims to estimate the current prevalence of obesity in Irish children, and to explore determinants of childhood obesity at an individual, family, and environmental level, with a specific focus on dietary patterns and physical activity. The secondary aim of the CCLaS study is to estimate average salt intake and examine BP distribution in Irish children.

## Methods

### Aims and Objectives

The CCLaS study aims to assess the current prevalence of overweight and obesity in Irish children, and explore risk factors at an individual, family, and environmental level in a sample of children 8-11 years of age in primary schools in Cork, Ireland.

### Primary Objectives

A primary objective is to assess the weight status and estimate the current prevalence of overweight and obesity using objectively measured height, weight, waist circumference, and skinfold thickness measurements in Irish children 8-11 years of age.

Another primary objective is to explore individual, family, and environmental factors associated with childhood overweight and obesity, with a specific focus on dietary patterns and objectively measured physical activity.

### Secondary Objectives

A secondary objective is to assess the average salt intake and distribution of BP in children 8-11 years old in Ireland.

### Study Population

The CCLaS study is a cross-sectional survey conducted in Cork, Ireland. Cork is located in the South West of Ireland, and Cork City has a population of 120,000. Mitchelstown is a rural area in Cork County with a population of >3000, and is located approximately 50 kilometers from Cork City. Information on primary schools in Cork City and Mitchelstown was obtained from the Department of Education and Skills website [42]. The website contains information on school name, location, gender mix, size, and disadvantaged status. Disadvantaged status is assigned to schools based on the sociodemographic and socioeconomic profile of the families whose children attend the school [42]. At the national level, one in five primary schools has disadvantaged status. However, nearly half of Cork City schools have disadvantaged status, with approximately 40% of primary school children in Cork City attending a disadvantaged school [42].

Special needs schools and schools without age eligible children were excluded from the sampling frame. All other primary schools in Cork City and Mitchelstown were included in the sampling frame. At the time of sampling, there were 51 primary schools with approximately 13,230 students in Cork City which met the sampling frame criteria. All 5 primary schools in Mitchelstown (with approximately 800 students) met the sampling frame criteria [42]. Children in 3rd and 4th classes (years 5 of 6 of enrollment into primary school) were the target population, as we wished to recruit children of a similar age to previously conducted Irish research [39].

### Sampling Method and Sample Size

The study aimed to recruit 1000 participants in order to estimate the prevalence of overweight and obesity in Irish children with a precision of ±2.7%, assuming a 26% prevalence rate of overweight and obesity within the study sample [43]. Allowing for a response rate of 70%, it was estimated that 1500 participants would need to be invited to partake in the study.

**Figure 1:**
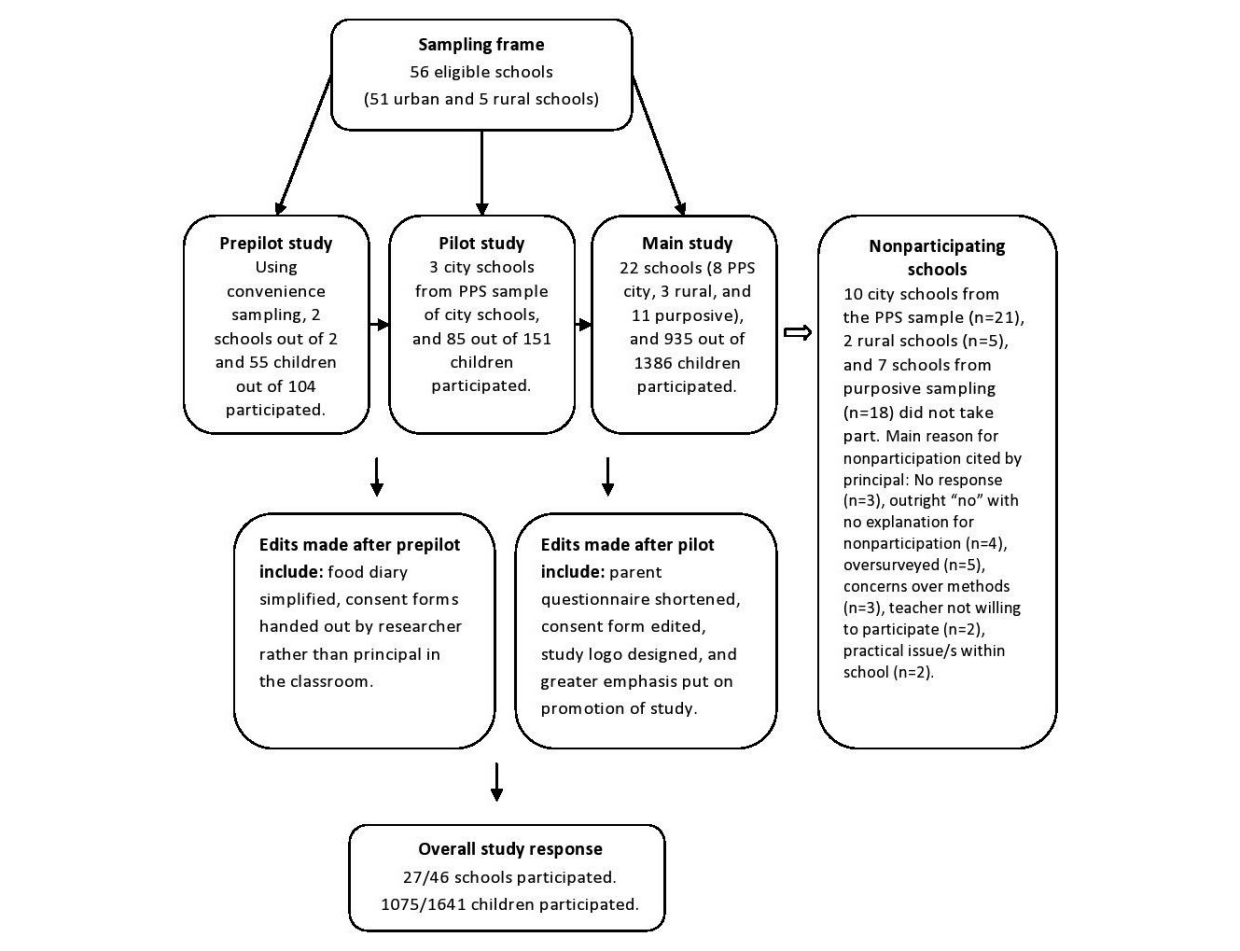
Flowchart of sampling and recruitment of schools and children in the Cork Children’s Lifestyle Study. PPS=probability proportionate to size.

For the prepilot study, 2 city schools were recruited using convenience sampling. For the pilot and main study, a probability proportionate to size (PPS) sampling strategy was used to select a random sample of primary schools in Cork City. The PPS sample of city schools was based on school size. A small school was defined as having <100 pupils, a medium school having 100-300 pupils, and a large school having >300 pupils. A complete sample of schools in Mitchelstown was invited to participate in the study. In order to achieve the sample size requirements, the schools not willing to participate in Cork City were replaced using a further purposive sampling strategy. The schools not willing to participate were replaced to represent the sampling frame population for: (1) school disadvantaged status, and (2) gender. As the recruitment of schools was undertaken over two consecutive school years, schools were sampled without replacement. All children in 3rd and 4th classes of participating primary schools were invited to participate in the study. Figure 1 shows a summary of the sampling and recruitment process.

### School and Participant Recruitment

The principals (school heads) of selected schools were sent an invitation letter, an information sheet, and a presentation containing study details. The principal was then contacted by telephone to arrange a face-to-face appointment with a study researcher to discuss the study. During the study meeting with the principal, the study aims, proposed methods, and study procedures were discussed. With the principals’ permission, the research team introduced the study to the 3rd and 4th class children of participating schools, and a parent/guardian information letter and consent form was given to each child to bring home. The children were advised to discuss the study with their parents/guardians, and to return the consent form to the school if they and their parents/guardians were willing to participate. The parent/guardian consent form was divided into 3 sections. The first section gave permission for the study child to participate in the study. The second section gave permission for a urine sample to be provided by the study child, and the third section gave permission for the urine sample to be stored by long term freezing.

### Data Collection Methods

#### Testing/Piloting

Prior to the main study, a prepilot study was conducted in two Cork City primary schools in April-May 2012, and a pilot study in 3 Cork City primary schools in May-June 2012. Overall, one hundred and forty children from 2 mixed gender schools, two boys’ schools, and one girls’ school were recruited to participate. The study piloting aimed to test practical research issues including the timing of procedures. The study methods and study documents including the food diary and questionnaires were also tested and assessed during piloting. Study documents, the study protocol, and standard operating procedures (SOP) were amended where necessary. Modified versions of the study documents are available from the lead author on request.

#### Schools and Classroom Procedures

The study researchers were advised to strictly adhere to the methods outlined in the study protocol and SOP during the fieldwork process. Within the classroom, each child was provided with a study pack which contained: (1) a child questionnaire, (2) a parent/guardian questionnaire, (3) a 3 day estimated food diary, (4) an accelerometer and instructions, and (5) a urine collection cup and instructions (where parent/guardian consent was granted). The research assistants were present for all classroom procedures and offered support and assistance where necessary. The children completed a self-reported questionnaire within the classroom, which was checked for completeness while on site. The accelerometers were described and placed on the nondominant wrist of each child. The 3 day estimated food diary was explained using a poster template of the food diary. The researchers explained how to fill in the food diary, and with assistance, that morning’s breakfast was completed by the children within the classroom. The children were informed how and what day to provide the urine sample, which was to be returned to the school once complete. The children were also instructed to return the parent/guardian questionnaire to the school once complete. A “pictogram” poster was placed in the classroom to remind children of the of study details they needed to recall.

### Questionnaire Data

Multimedia Appendix 5 outlines the individual, family, and environmental factors measured in each questionnaire (see Multimedia Appendices 1-3, and 5 for questionnaires and outline of factors). The questionnaires were developed based on previously tested and validated questions, with modification of some questions for the purposes of this study. Details of each questionnaire are described below.

### Principal Questionnaire

The principal of each participating school was asked to complete a questionnaire which included questions under 6 main headings: (1) demographics, (2) health curriculum, (3) school policy environment, (4) level of nutritional care, (5) provision of physical activity, and (6) parental/community support. This questionnaire has been used previously in a cross-sectional study in schools in Cork City [44].

### Child Questionnaire

The child questionnaire was developed using questions from the following sources: (1) Sport, Physical Activity and Eating Behavior: Environmental Determinants in Young People study [27], (2) Growing Up in Ireland (GUI) study [39], (3) Growing Up in Australia: The Longitudinal Study of Australian Children (LSAC) [45], (4) Child Heart and Health Study in England [46], and (5) Physical Activity for Older Children Questionnaire [47].

The child-reported questionnaire contained questions under 5 major headings: (1) background information, (2) your neighborhood, (3) food and diet, (4) sports and physical activity, and (5) hobbies and activities.

### Parent/Guardian Questionnaire

The parent/guardian questionnaire was developed using questions from a number of sources: (1) GUI study [39], (2) Survey of Lifestyle, Attitudes, and Nutrition in Ireland [48], (3) Avon Longitudinal Study of Parents and Children [49], (4) LSAC study [45], (5) National Survey of Children’s Dental Health [50], (6) Eating Among Teens Survey 1 [51], (7) Mitchelstown Cohort study [52], (8) Irish Census [53], (9) Child Feeding Questionnaire [54], (10) short version (self-administered) of the International Physical Activity Questionnaire [55], and (11) Warwick-Edinburgh Mental Well-being Scale [56].

The parent/guardian-reported questionnaire contained questions under 9 major headings: (1) study child’s birth factors, (2) study child’s current health, (3) study child’s exercise and physical activity, (4) study child’s hobbies and activities, (5) study child’s diet and dietary habits, (6) current parental health, (7) parental diet, (8) general family eating questions, and (9) family background.

### Dietary Intake

Dietary intake was assessed using a consecutive 3 day estimated food diary which was developed for the purposes of this study (see Multimedia Appendix 4 for template of food diary). Instructions to complete the food diary, including food atlas photographs [57] to aid portion size estimation, were located at the beginning of the food diary. Each day in the food diary was broken into six meal sections. Each meal section had a preassigned title: (1) breakfast, (2) morning snack, (3) lunch, (4) afternoon snack, (5) dinner, and (6) evening snack. There were six key questions to answer within each meal section: (1) time meal/snack was consumed, (2) location meal was consumed, (3) type of food or drink consumed, (4) quantity of food or drink consumed, (5) quantity leftover, and (6) cooking method used. The food diary was explained to the children in the classroom setting. First, the layout of the food diary was explained. Using a poster template, the children were shown how to fill in each meal section. The children were also shown how to use the food atlas photographs at the beginning of their food diary to help estimate portion size.

Once the food diary was explained in the classroom, the children were asked to fill in what they had for breakfast that morning. A member of the research team spent some time with each child to ensure that they understood what was involved. The children were advised to seek help from parents and teachers when filling in their food diary where possible. Detailed debriefing with the children occurred after the 3 day period using a prompt sheet and food atlas [57] in order to ensure completeness. Where necessary, additional information was sought from the children, especially where food or drink items were not recorded in detail. Food diary data will be analyzed using WISP version 4 (Tinuviel Software, Anglesey, UK). Output measures will include nutrient intake, individual food intake, and food group intake.

### Physical Activity

Free living physical activity was measured over a consecutive 7 day period using a validated tri-axial GENEActiv accelerometer [58,59]. The GENEActiv accelerometer is a small, light weight, waterproof device [60]. The manufacturer (Activinsights Limited) calibrated the units prior to the study commencing. The accelerometers were set to record data at 100Hz for 7 days using the “on button press” setting on the GENEActiv software version 2.2. The children were asked to wear the accelerometer all day and night over the 7 day period. They were informed only to remove the accelerometer for sports if their coach suggested it was necessary. The accelerometers were fitted on the wrist of the nondominant hand, and information on handedness was recorded by the research assistants. The accelerometers were downloaded in “.csv” and “.bin” format, and saved on hard drives. The data will be collapsed into 1 second and 1 minute epochs for data analysis. Output measures will include sedentary time, low, moderate, and vigorous activity. The classification thresholds for activity intensity will be defined using those outlined by Phillips et al, which were designed specifically for the GENEA accelerometer [59].

### Anthropometric and Blood Pressure Measurements

The anthropometric and BP measurements were taken by fully trained researchers using standard procedures. The researchers received training from an experienced research nurse and dietician prior to the study commencing. Retraining sessions occurred during the data collection period to ensure standard procedures were being employed during measurements. The data was also checked for measurement variability during the data collection period. The study equipment was calibrated prior to data collection, and monthly thereafter.

A summary of the anthropometric and BP measurements methods is described in Table 1. All measurements were taken in a sensitive manner in a private room or behind screens in each primary school. There were two children and at least two research assistants that remained in the room at all times. For the waist circumference and skinfold thickness measurements where two readings were taken, the mean value will be used for analysis. The children will be classified as normal weight, overweight, or obese using age and gender specific International Obesity Taskforce cut off points [61]. Mean systolic and diastolic BP will be calculated using the average of readings two and three.

**Table 1:** Summary of study methods.

Measure	Number of measures	Device	Method
Height	1	Leicester portable height stick	Measured to the nearest mm without shoes.
Weight	1	Tanita WB100MA mechanic scales	Measured to the nearest 0.1 kg without shoes and in light clothing.
Waist circumference	2	Nonstretch tape Seca 200 measuring tape	Measured to the nearest mm and located at the midpoint between the child’s lower rib margin line and the iliac crest.
Skinfold thickness (triceps)	2	Holtain Tanner/ Whitehouse skinfold calipers	Measured at the right hand side of the body to the last complete mm. The triceps was located on the posterior midline of the upper arm, over the triceps muscle, halfway between the acrosion process and olecranon process. The elbow was extended and relaxed for the measures.
Skinfold thickness (subscapular)	2	Holtain Tanner/ Whitehouse skinfold calipers	Measured at the right hand side of the body to the last complete mm. The subscapular was located on the diagonal line coming from the vertebral border to between 1 and 2 cm from the inferior angle of the scapulae.
Mid upper arm circumference	1	Nonstretch tape	Measured using a nonstretch tape to the nearest mm from the right arm while relaxed. The midpoint was located half ways between the top of the shoulder and the tip if the elbow.
BP	3	Omron M6	BP was measured from the right arm using a validated automatic oscillometric device [62,63]. The mid upper arm circumference determined cuff size. The cuff was placed approximately 2 cm above the crease of the elbow. The child was seated comfortably for at least 5 minutes prior to the first reading. BP was measured three times, with one minute between each measurement. Children were asked to remain quiet and to sit still while each reading was being taken. Systolic BP, diastolic BP, and pulse were recorded.
Accelerometer	7 consecutive days	GENEActiv	Accelerometers were set to record data at 100 Hz and was worn on nondominant hand for 7 days.
Estimated food diary	3 consecutive days		Children recorded everything they ate and drank for 3 days. Food diaries were fully debriefed by a trained researcher after the 3 day period.
Early morning spot urine sample	1		Children were asked to provide an early morning spot sample on a day, which corresponded to a food diary completion day.
24 hour urine sample	1		A subsample of children were asked to provide a 24 hour urine sample on a weekend day, which corresponded to a food diary completion day.

### Urine Samples

Only children whose parents provided consent for urine collection were provided with a urine collection cup and instructions. The children were asked to provide an early morning spot urine sample on a specified day, which corresponded to a food diary completion day. Where principals were agreeable, a subsample of children were asked to provide a 24 hour urine sample (n=100) on a weekend day, which corresponded to a food diary completion day. There were sixteen children from one of the prepilot schools that were asked to provide an early morning spot and 24 hour sample. The 24 hour samples provide an indication of average urine volume produced in a 24 hour period by the children. Osmolality testing was carried out on the 24 urine samples to determine urine concentration using a Micro-Osmometer Model 3300 in Cork University Hospital, Cork, Ireland. The hydration status of the children with 24 hour samples will be determined from the osmolality derived urine concentrations. All samples were analyzed for sodium, potassium, urea, and creatinine in the Biochemistry Department in the Mercy University Hospital, Cork, Ireland (Accredited Laboratory ISO-15189). All electrolytes were analyzed using the Abbott Architect c8000 (Abbott Laboratories). The methodology for sodium and potassium measurement used ion-selective electrodes, urea analysis was based on an enzymatic assay using urease, and creatinine was analyzed using the kinetic alkaline picrate method. Where consent was provided, a 2 ml aliquot urine sample was frozen in a secure, password protected freezer.

### Ethics and Ethical Issues

Ethical approval for the CCLaS study was obtained from the Clinical Research Ethics committee of the Cork Teaching Hospitals, Cork, Ireland. Only children with parent/guardian informed consent participated in the study, and parents/guardians were free to withdraw their children from the study at any point. Feedback on the physical measurements was provided to all parents of participating children in the form of a letter. The parents of children with high BP or morbid obesity were advised to consult their general practitioner, and a general practitioner letter was enclosed with the feedback. A consultant pediatrician and a consultant in general internal medicine and nephrology from the Mercy University Hospital, Cork, Ireland provided advice on any high or unusual readings prior to feedback being provided to parents.

### Data Processing and Quality Assurance

Comprehensive data cleaning was undertaken. First, all data were checked for outliers. There was 10.04% (108/1075) of the data that was then randomly selected and rechecked for errors. Out of the 39,999 questionnaire data points checked, 139 errors were found and corrected. An error rate was then calculated (0.35% for questionnaire data). Missing data will be accounted for during data analysis either by data imputation or by creating missing data categories. Imbalances in the study sample will be accounted for using sampling weights. A standardized codebook will be generated to ensure standard definitions and cut off points are used during analysis.

### Analysis Plan

The data will be analyzed using the statistical software package Stata 12 IC (StataCorp LP). All necessary statistical assumptions will be tested prior to data analysis. Basic descriptive statistics will be used to describe the study population, and will provide prevalence estimates of overweight and obesity. Basic descriptive statistics will also be used to explore BP distribution. Descriptive findings will be stratified by gender. Crude and adjusted multivariate analysis will be conducted to assess the association between outcome variables and possible determinants. A multilevel approach will be adopted to examine obesity and possible group level determinants (individual, family, and environment). Latent class analysis will be conducted to identify subtypes of Irish children with respect to their diet, physical activity, lifestyle choices, and obesity risk.

## Results

Data collection was undertaken between April 2012 and June 2013. Preliminary data analysis is underway. It is anticipated that the results of the CCLaS study will be available in late 2014.

## Discussion

### Lessons Learned During the Pilot Studies

The pilot studies provided valuable insight into a number of practical and methodological issues. The practical and operational issues encountered included timing, obtaining an adequate response rate, and increasing awareness of the study in the local community. Obtaining a principal’s consent for a school to take part in the study took longer than anticipated, especially when teachers, board of management committees, and parent associations were consulted. In the main study, greater lengths of time were allowed when approaching schools to participate in the study. A relatively low response rate from parents and children was obtained during the piloting phase of the study. A possible explanation for this is that the piloting phase of the study was undertaken close to the summer holidays. However, for the main study a number of methods were used to encourage a greater response rate. The children were given a longer period of time to return the consent forms, a study logo was designed, and researchers wore study t-shirts with the logo when introducing the study in order to be more child friendly. Numerous phases of promotion of the study were also undertaken, with articles being written in local newspapers and letters being sent to local health and community organizations promoting the study. Study posters were also placed in shops and businesses throughout Cork City and Mitchelstown.

Methodological issues were also encountered, especially in terms of study document design. The original consent form was too complicated, and as a result was not being completed correctly by parents. In some cases it was difficult to decide if a parent was providing consent or not. Therefore, the consent form was made clearer and easier to complete. The parent questionnaire appeared to be too long, and this may have acted as a disincentive for parents to complete later sections in the questionnaire. For the main study, a number of questions were removed, and the questions of utmost importance were located at the start of the questionnaire. On the cover page of the questionnaire, parents were informed of the aim of the questionnaire and of the anticipated length of time needed to complete all of the questions. The food diary used in the pilot study was too complicated for children 8-11 years old to understand and fill in completely. As a result, this made the debrief process difficult. The food diary was made more child-friendly by changing the layout, reducing the number of questions asked about each meal, and by including a number of photographs from the food atlas [57] at the beginning of the food diary to aid portion size estimation.

### Recruitment Issues

Recruitment from schools is a difficult, multilevel process involving principals, teachers, parents, and their children [64]. Some research suggests that recruiting schools to participate in studies is becoming increasingly difficult, with nonresponse within schools becoming increasingly evident [65-67]. The CCLaS study aimed to collect data from a predominantly urban location (Cork City) and from one rural location (Mitchelstown). It was intended that an equal proportion of girls and boys would be recruited, and that the proportion of children attending disadvantaged versus nondisadvantaged schools would represent the sampling frame. During the study, recruitment of schools proved difficult, and further purposive sampling was necessary to achieve sample size requirements. A greater proportion of boys participated, and this is likely due to the nature of the study methods used. Boys only schools appeared to be more interested than girls only schools in the physical activity and accelerometer aspects of the study, and were interested to participate for this reason. On the contrary, the principals of nonparticipating girls only schools appeared more concerned about the anthropometric aspect of the study, and some principals expressed concerns over the sensitivity and possible long-term implications of measuring children.

Disadvantaged schools were more difficult to recruit than nondisadvantaged schools. Some school principals expressed concerns over the study methods, especially regarding children providing a urine sample. There were three principals from disadvantaged schools that agreed to take part in the study only on the condition that urine samples were not collected from the children in their school. School principals reported a variety of other reasons for not partaking. These include the low literacy of parents whose children attend the school, parents being suspicious of the study or study methods, the school being too busy, and other schools gave an outright “no” with no explanation for nonparticipation. Research fatigue in Cork City schools was also evident, with a number of nonparticipating schools reporting they had just taken part in a different study or found studies overly time consuming. The proximity of city schools to local research institutions is a likely explanation for research fatigue, and thus, further school based studies require carefully designed recruitment strategies.

### Strengths

The sample size is relatively large, and represents 1075 children out of approximately 3350 eligible children in the overall sampling frame. A predominant strength of this study is the depth of data on lifestyle, diet, and physical activity data collected at an individual, family, and school level which will allow for in-depth exploration on the potential determinants of childhood overweight and obesity. This is one of the first studies in Europe designed to collect such data. A number of objective anthropometric measurements were taken to describe weight status. The study collected objectively measured physical activity data in free living conditions over a 7 day period. The corresponding physical activity questionnaire data will provide valuable information of the context of physical activity behaviors and patterns. Seasonality will be accounted for, as the data was collected throughout the school year (October-June). The thoroughly debriefed 3 day estimated food diaries provide comprehensive data on dietary intake patterns and behaviors. This is the first study, to our knowledge, in Ireland to provide objective estimates of salt intake from spot and 24 hour urine samples, and to assess the distribution of BP in a large sample of Irish children.

### Limitations

There are a number of limitations to the study. A relatively low response rate was obtained from the original sample of city schools, though the desired sample size was achieved using purposive sampling. However, some response bias may have been introduced into the study. Information on nonresponding children is not available. As the food dairies are self-reported, some misreporting and nonreporting may have occurred. However, the food diaries were thoroughly debriefed by a trained researcher, though this may have resulted in some reporting bias of dietary intake. A 3 day food diary may not be representative of habitual dietary intake. Some response bias may have been introduced into the child questionnaire responses, as they were completed in a classroom setting, though children were encouraged to complete the questionnaires independently. As this study is cross-sectional in nature, no causal inference can be implied.

### Conclusions

This study aims to estimate the current prevalence of overweight and obesity in 8-11 year old Irish children. The research from the CCLaS study will explore the individual, family, and environmental correlates of childhood obesity, and will identify clusters of Irish children in relation to their dietary and physical activity patterns and lifestyle choices. The distribution and determinants of children’s salt intake and BP will also be analyzed as part of this study. To date, there are no reliable data on the average salt intake or distribution of BP in Irish children. Valuable comparisons with findings at an Irish, European, and International level will be made. In particular, CCLaS study findings will be compared to results from the GUI study, which is a national longitudinal study of children in the Republic of Ireland. The CCLaS study aims to highlight the modifiable social, economic, and cultural dimensions of childhood obesity. It is anticipated that this will highlight areas of action for policymakers, planners, and developers with a responsibility for addressing childhood obesity and creating sustainable healthy environments.

## Multimedia Appendix 1

Principal questionnaire from the Cork Children's Lifestyle Study (CCLaS).

## Multimedia Appendix 2

Child questionnaire from the Cork Children’s Lifestyle Study (CCLaS).

## Multimedia Appendix 3

Parent questionnaire from the Cork Children’s Lifestyle Study (CCLaS).

## Multimedia Appendix 4

Food diary template from the Cork Children’s Lifestyle Study (CCLaS).

## Multimedia Appendix 5

Individual, family and environmental factors measured by the CCLaS questionnaires.

## Abbreviations

BP: blood pressure
CCLaS: Cork Children’s Lifestyle Study
GUI: Growing Up in Ireland study
LSAC: The Longitudinal Study of Australian Children
PPS: probability proportionate to size
SOP: standard operating procedures
