# Supplementary material for: Diet, Physical Activity, Lifestyle Behaviors, and Prevalence of Childhood Obesity in Irish Children: The Cork Children’s Lifestyle Study Protocol
Source: JMIR Res Protoc. 2014 Aug 19;3(3):e44. doi: 10.2196/resprot.3140 (PMC4147704; doi:10.2196/resprot.3140)
Supplement: Supplementary file 1 [file resprot_v3i3e44_app1.pdf]

PRINCIPALS QUESIONNAIRE

CORK CHILDREN'S LIFESTYLE STUDY

School ID ☐☐☐ - ☐☐

This survey is designed to measure the school health environment of primary schools. Your answers will help us to identify best practice, make policy recommendations and specify future areas of research. Information provided relating to your specific school will be kept confidential. If you do not know the answer, please mark 'don't know' rather than guess.

Demographic Information

1. Please tick to indicate your sex:

☐ Male

☐ Female

2. Please tick to indicate what type of school you work at *(tick only one)*:

☐ Mixed national school

☐ Girls national school

☐ Boys national school

☐ Private primary school

3. How many children are attending your school this year?

Girls _____
Boys _____

4. How many members of staff do you have this year?
Male teachers ____
Female teachers ____
Administrative staff ____
Other non-teaching staff ____

Health Curriculum

5. Within the 'Social, Personal and Health Education (SPHE)' area of the national curriculum, does your school include a strand on Food and Nutrition?

☐ Yes

☐ No

☐ Don't know

5a. On average, how many **hours per term** do the children spend in classes on Food and Nutrition in the SPHE strand of the national curriculum?
Infants ____________hours per term
Primary students ____________hours per term

The School Environment

6. Are you aware of the Health Services Executive (HSE) Health Promoting Schools Programme?

☐ Yes

☐ No

☐ Don't know

6a. Is your school involved in the HSE Health Promoting Schools Programme?

☐ Yes

☐ No

☐ Don't know

6b. If so, how long have you been involved in the HSE Health Promoting Schools Programme?

________Years ________months

7. Does your school have a healthy school policy (also known as a wellness policy)?

☐ Yes

☐ No

☐ Don't know

(*if yes, please continue on to question7a; if no or don't know, please go to question 8)*

7a. Please **include a copy of your healthy school policy.**

7b. Does it include a written healthy food policy with an available document?

☐ Yes

☐ No

☐ Don't know

7c. Please indicate who was involved in the creation of your healthy school policy (*tick all that apply):*

- ☐ Pupils
- ☐ Teaching staff
- ☐ Non-teaching staff
- ☐ Parents
- ☐ Other community members (eg church, businesses, health practitioners)
- ☐ Don't know

8. Over the past school year, has your school led or participated in any health promotion activities?

- ☐ Yes
- ☐ No
- ☐ Don't know

8a. Please indicate if your school has been involved in any health promotion activities listed below (*tick all that apply):*

- ☐ Awareness-raising activities or campaigns around particular health issues
- ☐ Special lectures or assemblies with a health promotion theme
- ☐ Visitors brought in to talk about health-related issues
- ☐ Projects involving the HSE Health Promotion unit
- ☐ Sports days
- ☐ Nutrition campaigns
- ☐ Don't know
- ☐ Not applicable

9. Are there any particular members of staff who have spontaneously organized or proposed an initiative on student diet or exercise in the past year?

- ☐ Yes
- ☐ No
- ☐ Don't know

10. Have any attempts been made to integrate issues of diet and exercise into the school environment?

- ☐ Yes
- ☐ No
- ☐ Don't know

School Food

11. Can pupils buy food on the school premises?

- ☐ Yes
- ☐ No
- ☐ Don’t know

12. Does your school have Designated Disadvantaged Status?

- ☐ Yes
- ☐ No
- ☐ Don’t know

12a. Does your school provide lunches through the Social and Family Affairs School Meals Scheme?

- ☐ Yes
- ☐ No
- ☐ Don’t know

13. Does your school serve breakfast?

- ☐ Yes
- ☐ No
- ☐ Don’t know

14. Does your school serve lunch?

- ☐ Yes
- ☐ No
- ☐ Don’t know

15. Is food prepared on-site?

- ☐ Yes
- ☐ No
- ☐ Don’t know

16. Does your school have kitchen facilities?

- ☐ Yes
- ☐ No
- ☐ Don’t know

17. Does your school provide options for healthy choices in school food?

- ☐ Yes
- ☐ No
- ☐ Don't know

18. Does your school have any of the following on the school premises?

	Yes	No	Don't know
Canteen or cafeteria			
Tuck shop or snack bar			
Vending machines			
Outside food vendors such as ice-cream vans or other snack facilities			

19. Please indicate if the following foods are available to students at your school *(tick all that apply)*:

- ☐ Crisps
- ☐ Nuts
- ☐ Biscuits
- ☐ Yogurt
- ☐ Chips
- ☐ Granola or cereal bars
- ☐ Chocolate
- ☐ Fruit (fresh, canned or dried)
- ☐ Vegetables
- ☐ Sweets
- ☐ Cakes
- ☐ Milk
- ☐ Water
- ☐ Fizzy drinks
- ☐ Fruit juices
- ☐ Other
- ☐ Not applicable

20. How many times in the current school year has your school been involved in competitive food sales: e.g., selling food as part of a fundraising effort ___________

21. Please indicate which of the following statements is true *(tick only one)*:

- ☐ Some of my staff uses food and/or beverages as incentives or rewards for students.
- ☐ None of my staff use food and/or beverages as incentives or rewards for students.

22. Please indicate which of the following statements is true *(tick only one)*:

- ☐ My school allows students to consume food and beverages outside of allocated meal times.
- ☐ My school does not allow students to consume food and beverages outside of allocated meal times.

Physical Activity

23. Does your school have any after school sports teams?

- ☐ Yes
- ☐ No
- ☐ Don’t know

24a. Please indicate if your school has any of the following after school sports teams *(tick all that apply)*:

- ☐ Hurling
- ☐ Rugby
- ☐ Football/soccer
- ☐ Gaelic football
- ☐ Basketball
- ☐ Netball
- ☐ Hockey
- ☐ Tennis
- ☐ Badminton
- ☐ Cricket
- ☐ Gymnastics
- ☐ Other
- ☐ Not applicable

23b. How many students participate in these sports teams? __________

24. Does your school provide any other after school activities other than sports teams?

- ☐ Yes
- ☐ No
- ☐ Don’t know

24a. Please indicate if your school has any of the following after school activities *(tick all that apply)*:

- ☐ Dance
- ☐ Drama
- ☐ Music
- ☐ Art
- ☐ Games (like chess club)
- ☐ Languages
- ☐ Religion
- ☐ Science
- ☐ Environment club
- ☐ Other academic-related club
- ☐ Other
- ☐ Not applicable

24b. How many students participate in these other after school activities? __________

25. Does your school provide students with outdoor break time?

- ☐ Yes
- ☐ No
- ☐ Don’t know

25a. How many hours per day?__________ Hours __________ minutes

26. Please indicate if your school has the following facilities available to students *(tick all that apply)*:

- ☐ Gymnasium
- ☐ Playground
- ☐ Bicycle racks
- ☐ Sports fields
- ☐ Swimming pool
- ☐ Basketball hoop
- ☐ Badminton court
- ☐ Tennis court
- ☐ Dance studio

Parental and Community Support

27. Does your school have a Parents' Association?

- ☐ Yes
- ☐ No
- ☐ Don't know

27a. How active is the Parents' Association?

- ☐ Not active at all;
- ☐ Somewhat active;
- ☐ Active;
- ☐ Very active

27b. How many parents are active in your Parents' Association? ___________

28. Does your school run any of the following classes specifically for parents and/or the community *(tick all that apply)*?

- ☐ Cooking
- ☐ Art
- ☐ Music
- ☐ Parent and child groups or activities
- ☐ Exercise/physical activity/sports
- ☐ Languages (including English)
- ☐ Continuing education
- ☐ Vocational training
- ☐ Other
- ☐ Not applicable

28a. How many parents and/or members of the community are involved in these classes? ___________

29. Have any parents in the past year brought up issues relating to students' diet?

- ☐ Yes
- ☐ No
- ☐ Don't know

29a. Have any parents requested in the past year that more fruits and vegetables be served at school?

- ☐ Yes
- ☐ No
- ☐ Don't know

30. Have any parents in the past year brought up issues relating to physical activity?

- ☐ Yes
- ☐ No
- ☐ Don't know

30a. Have any parents requested in the past year that students participate in more physical activity at school (such as more physical education classes?)

- ☐ Yes
- ☐ No
- ☐ Don't know
