# Supplementary material for: Diet, Physical Activity, Lifestyle Behaviors, and Prevalence of Childhood Obesity in Irish Children: The Cork Children’s Lifestyle Study Protocol
Source: JMIR Res Protoc. 2014 Aug 19;3(3):e44. doi: 10.2196/resprot.3140 (PMC4147704; doi:10.2196/resprot.3140)
Supplement: Supplementary file 2 [file resprot_v3i3e44_app2.pdf]

Cork Children's Lifestyle Study – Child Questionnaire

Cork Children's Lifestyle Study

Official use | C | C | L | S | | | | | S | C | H | | |

A. BACKGROUND INFORMATION

Please tick one box

A.1. Are you a boy or a girl: **SOURCE: SPEEDY**

☐ Boy ☐ Girl

A.2. How old are you? ☐ **SOURCE: SPEEDY**

A.3. Do you have brothers or sisters? (GUI)

☐ Yes ☐ No

A.4. How would you describe your health? **SOURCE: DEVELOPED BY CCLaS RESEARCH TEAM**

☐ Excellent ☐ Fair

☐ Good ☐ Poor

A.5. How would you describe yourself? **SOURCE: GUI**

☐ Very skinny ☐ A bit overweight

☐ A bit skinny ☐ Very overweight

☐ Just the right size

B. YOUR NEIGHBOURHOOD

B.1. Thinking about where you live...Do you **like** the area you live in? **SOURCE: LSAC**

☐ A lot ☐ Not very much

☐ Quite a lot ☐ Not at all

B.2. Is there **a playground or park** near where you live? **SOURCE: LSAC**

☐ Yes ☐ No

B.3. Are there places for children to **play safely** near your home? **SOURCE: LSAC**

☐ Yes ☐ No

B.4. Do you **feel safe** in your neighbourhood? **SOURCE: LSAC**

☐ Yes

☐ Sometimes yes, sometimes no

☐ No

B.5. Is there **a garden** at your family home? **SOURCE: DEVELOPED BY CCLaS RESEARCH TEAM**

☐ Yes ☐ No

B.6. How often do your friends play at **your home**? [Include relatives of your own age if you count them as friends]. **SOURCE: LSAC**

☐ A few times a week ☐ A few times a year

☐ About once a week ☐ Never

☐ About once a month

B.7. How often do you play at **your friend's homes**? [Include relatives of your own age if you count them as friends]. **SOURCE: LSAC**

☐ A few times a week ☐ A few times a year

☐ About once a week ☐ Never

☐ About once a month

C. FOOD AND DIET

C.1. How many days per week do you eat **breakfast** before school? **SOURCE: GUI**

☐ Everyday ☐ Most days ☐ Never

If you answered Everyday Skip to Question C.3.

C.2. If most days or never, what is the reason why you skip breakfast? **[Please tick one box] SOURCE: DEVELOPED BY CCLaS RESEARCH TEAM**

- ☐ I don't like breakfast
- ☐ No one in my family eats breakfast
- ☐ I don't have time in the morning to eat breakfast
- ☐ There are no breakfast foods in my house
- ☐ Other ______

C.3. How often do you **add salt** to food while at the table? **SOURCE: SLÁN**

☐ Everyday ☐ Most days ☐ Never

C.4. What is your favourite snack? ______ **SOURCE: CHASE**

C.5. How often do you eat your favourite **snack**? **SOURCE: DEVELOPED BY CCLaS RESEARCH TEAM**

- ☐ Everyday
- ☐ 1-3 times a week
- ☐ 4-6 times a week
- ☐ Less than once a week

C.6. What is your favourite drink? ______ **SOURCE: CHASE**

C.7. How often do you drink your favourite **drink**? **SOURCE: DEVELOPED BY CCLaS RESEARCH TEAM**

- ☐ Everyday
- ☐ 1-3 times a week
- ☐ 4-6 times a week
- ☐ Less than once a week

D. SPORTS AND PHYSICAL ACTIVITY

D.1. Physical activity in your spare time:
Have you done any of the following activities in the **past 7 days** [last week]? If yes, how many times? **[Please tick one box per row] SOURCE: PAC_Q**

	No	1-2 times	3-4 times	5-6 times	7 times or more
Skipping	☐	☐	☐	☐	☐
Rowing/ canoeing	☐	☐	☐	☐	☐
Tag (chasing)	☐	☐	☐	☐	☐
Walking for exercise	☐	☐	☐	☐	☐
Cycling	☐	☐	☐	☐	☐
Jogging or running	☐	☐	☐	☐	☐
Swimming	☐	☐	☐	☐	☐
Rounders	☐	☐	☐	☐	☐
Dance	☐	☐	☐	☐	☐
Hockey	☐	☐	☐	☐	☐
Volleyball	☐	☐	☐	☐	☐
Basketball	☐	☐	☐	☐	☐
Soccer	☐	☐	☐	☐	☐
Football (GAA)	☐	☐	☐	☐	☐
Hurling/ camogie	☐	☐	☐	☐	☐
Rugby	☐	☐	☐	☐	☐
Tennis	☐	☐	☐	☐	☐
Judo/Taekwondo/Karate	☐	☐	☐	☐	☐
Other (give name) ________	☐	☐	☐	☐	☐
Other (give name) ________	☐	☐	☐	☐	☐

D.2. In the **last 7 days**, how many physical education [PE] classes did you have? **SOURCE: DEVELOPED BY CCLAS RESEARCH TEAM**

☐ 0 ☐ 1 ☐ 2 ☐ 3 ☐ 4 ☐ 5 or more

D.3. In the **last 7 days**, during your physical education [PE] classes, **how often were you very active** [playing hard, running, jumping, throwing]? **[Please tick one box] SOURCE: PAC_Q**

☐ I don't do PE ☐ Quite often

☐ Hardly ever ☐ Always

☐ Sometimes

D.4. In the **last 7 days**, what did you do most of the time at **morning break? [Please tick one box] SOURCE: PAC_Q**

☐ Sat down (talking, reading, doing school work)

☐ Stood around or walked around

☐ Ran or played a little bit

☐ Ran around and played quite a bit

☐ Ran and played hard most of the time

D.5. In the **last 7 days**, what did you normally do at **lunch break [besides eating lunch]**? **[Please tick one box] SOURCE: PAC_Q**

☐ Sat down (talking, reading, doing school work)

☐ Stood around or walked around

☐ Ran or played a little bit

☐ Ran around and played quite a bit

☐ Ran and played hard most of the time

D.6. In the **last 7 days**, on how many days **right after school**, did you do sports, dance, or play games in which you were very active? **[Please tick one box] SOURCE: PAC_Q**

☐ None

☐ 1 time last week

☐ 2 or 3 times last week

☐ 4 times last week

☐ 5 times last week

D.7. In the **last 7 days**, on how many **evenings** did you do sports, dance, or play games in which you were very active? **[Please tick one box] SOURCE: PAC_Q**

☐ None

☐ 1 time last week

☐ 2 or 3 times last week

☐ 4 or 5 times last week

☐ 6 or 7 times last week

D.8. On the **last weekend**, how many times did you do sports, dance, or play games in which you were very active? **[Please tick one box] SOURCE: PAC_Q**

☐ None

☐ 1 time last week

☐ 2 or 3 times last week

☐ 4 or 5 times last week

☐ 6 or 7 times last week

D.9. Which **one** of the following describes you best for the **last 7 days**? [Physical things, e.g. played sports, went running, swimming, bike riding, did aerobics]. Read all **five** statements before deciding on the **one** answer that describes you. **[Please tick one box] SOURCE: PAC_Q**

☐ All or most of my time was spent doing things that involve little physical effort

☐ I sometimes (1-2 times) did physical things in my free time

☐ I often (3-4 times) did physical things in my free time

☐ I quite often (5-6 times) did physical things in my free time

☐ I very often (7 or more times) did physical things in my free time

D.10. Mark **how often** you did physical activity [like playing sports, games, doing dance, or any other physical activity] for **each day last week**. **SOURCE: PAC_Q**

	None	Little bit	Medium	Often	Very often
Monday	☐	☐	☐	☐	☐
Tuesday	☐	☐	☐	☐	☐
Wednesday	☐	☐	☐	☐	☐
Thursday	☐	☐	☐	☐	☐
Friday	☐	☐	☐	☐	☐
Saturday	☐	☐	☐	☐	☐
Sunday	☐	☐	☐	☐	☐

D.11. Were you **sick last week**, or did anything prevent you from doing your normal physical activities? **[Please tick one box] SOURCE: PAC_Q**

☐ Yes

☐ No

☐ If yes what prevented you ____________________

E. HOBBIES, ACTIVITIES & PETS

E.1. Which of the following things do you have at **home**? [Please place a tick in the box for each thing you have at home. Leave the box empty for things you don't have.] **SOURCE: SPEEDY**

- ☐ More than one car [or van]
- ☐ A home computer
- ☐ A games console [such as Xbox, Playstation]
- ☐ An active games console [such as Nintendo Wii]

E.2. Do you have any of these **in your bedroom**? [Please place a tick in the box for each thing you have in your bedroom. Leave the box empty for things you don't have.] **SOURCE: SPEEDY**

- ☐ A television
- ☐ A DVD or video player
- ☐ A home computer
- ☐ A games console [such as an Xbox or Playstation]
- ☐ An active games console [such as Nintendo Wii]
- ☐ None of these

E.3. How often do you play **computer games and games console (such as Xbox, PlayStation)**? **[Please select one answer] SOURCE:DEVELOPED BY CCLaS RESEARCH TEAM**

- ☐ Never
- ☐ 1 - 2 days per week
- ☐ 3 - 4 days per week
- ☐ Nearly everyday

E.4. How often do you play the **active games console** [such as Nintendo Wii]? **SOURCE: DEVELOPED BY CCLaS RESEARCH TEAM**

☐ Never

☐ 1 - 2 days per week

☐ 3 - 4 days per week

☐ Nearly everyday

E.5. How much time do you spend **watching television** each day? **SOURCE: DEVELOPED BY CCLaS RESEARCH TEAM**

☐ None

☐ Less than one hour

☐ Between 1 and 3 hours

☐ Between 3 and 5 hours

☐ Over 5 hours

E.6. How often do you **get homework**? **SOURCE: GUI**

☐ Never

☐ 1 - 2 days per week

☐ 3 - 4 days per week

☐ Almost everyday

E.7. How much time do you spend doing **homework** each day? **SOURCE: DEVELOPED BY CCLaS RESEARCH TEAM**

☐ None

☐ Less than one hour

☐ Between 1 and 3 hours

☐ Between 3 and 5 hours

☐ Over 5 hours

E.8. What is your favourite hobby or activity? **SOURCE: GUI**

E.9. Is there a pet in your family? **SOURCE: GUI**

☐ Yes ☐ No

E.10. If yes, what pets do you have? **[Tick all that apply]** **SOURCE: GUI**

☐ Cat

☐ Dog

☐ Goldfish

☐ Rabbit

☐ Other [Please write down]

E.11. If your family has a dog, do you walk the dog? **SOURCE: DEVELOPED BY CCLaS RESEARCH TEAM**

☐ Yes ☐ Sometimes ☐ No

Thanks for all your help!
