# Supplementary material for: Diet, Physical Activity, Lifestyle Behaviors, and Prevalence of Childhood Obesity in Irish Children: The Cork Children’s Lifestyle Study Protocol
Source: JMIR Res Protoc. 2014 Aug 19;3(3):e44. doi: 10.2196/resprot.3140 (PMC4147704; doi:10.2196/resprot.3140)
Supplement: Supplementary file 3 [file resprot_v3i3e44_app3.pdf]

Cork Children's Lifestyle study (CCLaS)

Parent Questionnaire

(To be filled out by the parent/guardian of the study child)

This questionnaire is part of the Cork Children's Lifestyle Study that you have consented for your child to take part in. It has been designed to examine the lifestyle and health of both you and your child. Questions included examine birth factors, physical activity levels and hobbies of your child. Questions specific to the parent/ guardian include those on current health, the general family setting, physical activity and dietary factors.

Please attempt to answer every question. It should take about 20 minutes to fill in this questionnaire.

Your answers will be treated as strictly confidential and will be used only for the purposes of this study. This questionnaire can be returned in the envelope provided within the blue study folder your child has been provided with and we will collect it from your child's school.

If you would rather have the questionnaire administered by telephone, please contact the research team using the contact details below and we can arrange this.

Thank you for taking the time to provide this information. Your input will provide valuable information to the study.

Yours sincerely,

Eimear Keane,
Department of Epidemiology and Public Health,
Western Gate Building,
University College Cork
Tel:
Email:

RELATIONSHIP TO STUDY CHILD:

Q1. Are you the child's: **SOURCE: DEVELOPED BY CCLaS RESEARCH TEAM**

- ☐ Mother
- ☐ Father
- ☐ Other (Please Specify) [ ]

A. STUDY CHILD'S BIRTH FACTORS

A.1. If known, how much did your child weigh at birth? **SOURCE: GUI**

[ ][ ] Pounds [ ][ ] Ounces **OR** [ ][ ] Kilos [ ][ ][ ] Grams ☐ Don't Know

A.2. If known, was your child born late, on time or early? **SOURCE: GUI**

- ☐ Late Birth (42 weeks or more)
- ☐ On Time (37-40 weeks)
- ☐ Somewhat Early (33-36 weeks)
- ☐ Very Early (32 weeks or less)
- ☐ Don't Know

A.3. If known, what was the mode of delivery? **SOURCE: GUI**

- ☐ Normal Birth
- ☐ Vacuum Assisted Birth
- ☐ Forceps Assisted Birth
- ☐ Elective Caesarean
- ☐ Emergency Caesarean
- ☐ Don't Know
- ☐ Other [ ]

A.4. Was your child ever breastfed? **SOURCE: GUI**

- ☐ Yes
- ☐ No
- ☐ Don't Know
- ☐ Can't Remember

A.5. For how many months or weeks was your child breastfed? **SOURCE: GUI**

[ ][ ] Months **OR** [ ][ ] Weeks **OR** [ ][ ] Days

- ☐ Don't Know
- ☐ Can't Remember

B. STUDY CHILDS CURRENT HEALTH

B.1. In general, how would you describe your child's health in the past year? **SOURCE: GUI**

- ☐ Very Healthy, no problems
- ☐ Healthy, but with a few minor problems
- ☐ Sometimes quite ill
- ☐ Almost always unwell

B.2. Does your child have any ongoing chronic physical or mental health problem, illness or disability such as Asthma, ADHD etc? **SOURCE: GUI**

- ☐ Yes
- ☐ Don't Know
- ☐ No

<u>If No, please skip to question B.6.</u>

B.3. What is the nature of this problem, illness or disability? Please describe as fully as possible. (Please record **diagnosis, not symptoms** of the problem) **SOURCE: GUI**

B.4. How old was your child when he/she was diagnosed with this problem, illness or disability? **SOURCE: GUI**

☐ Months Old **OR** ☐ Years Old

B.5. Is your child hampered in his/her daily activities by this problem, illness or disability? **SOURCE: GUI**

☐ Yes, severely ☐ Yes, to some extent ☐ No

B.6. Do you think your child is: **SOURCE: GUI**

☐ Very underweight
☐ Moderately underweight
☐ Slightly underweight
☐ About the right weight
☐ Slightly overweight
☐ Moderately overweight
☐ Very overweight
☐ Don't know

B.7. Does your child go to bed at a regular time? **SOURCE: LSAC**

☐ Always
☐ Usually
☐ Sometimes
☐ Rarely
☐ Never

B.8. On **normal school days,** what time in the morning does your child usually **wake up? SOURCE: ALSPAC**

hours ☐☐ **minutes** ☐☐ am

B.9. On **normal school days**, what time in the evening does your child usually **go to bed? SOURCE: ALSPAC**

hours ☐☐ **minutes** ☐☐ pm

B.10. On **weekends**, what time in the morning does your child usually **wake up? SOURCE: ALSPAC**

hours ☐☐ **minutes** ☐☐ am

B.11. On **weekends**, what time in the evening does your child usually **go to bed? SOURCE: ALSPAC**

hours ☐☐ **minutes** ☐☐ pm

B.12. How often does your child brush his/her teeth (or have them brushed for him/her)? **SOURCE: NATIONAL SURVEY OF CHILDREN'S DENTAL HEALTH**

- ☐ My child's teeth are not usually brushed
- ☐ Less than once a day (e.g. every second day, once a week)
- ☐ Once a day
- ☐ Twice a day
- ☐ More than twice a day

C. STUDY CHILD'S EXERCISE AND PHYSICAL ACTIVITY

C.1. How many times in the **past 7 days** has your child done at **least 20 minutes of hard exercise,** hard enough to make him / her breathe heavily and make his / her heart beat **faster**? (Hard exercise includes, for example, playing football, jogging, or fast cycling). Include time in physical education class. **SOURCE: GUI**

☐ None ☐ 1 to 2 days ☐ 3 to 5 days ☐ 6 to 7 days

C.2. How many times in the **past 7 days** has your child done at **least 20 minutes of light exercise** that was **not** hard enough to make him / her breathe heavily and make his / her heart beat fast? (Light exercise includes, for example walking or slow cycling). Include time in physical education class. **SOURCE: GUI**

☐ None ☐ 1 to 2 days ☐ 3 to 5 days ☐ 6 to 7 days

C.3. How does your child usually (a) go to school and (b) come home from school? **SOURCE: GUI**

(Tick one box in Col A and B)

	**A. Going**	**B. Coming Home**
He/ she walks	☐	☐
By public transport	☐	☐
By public transport and walking	☐	☐
School bus/coach	☐	☐
By car	☐	☐
Rides a bicycle	☐	☐
Other (please describe) ______	☐	☐

C.4. How **long** does it take your child (a) to go to school (b) to come home from school? **SOURCE: GUI**

(Tick one box in Col A and B)

	**A. Going**	**B. Coming Home**
Less than 5mins	☐	☐
5 mins - less than 10 mins	☐	☐
10 mins - less than 20 mins	☐	☐
20 mins - less than 30 mins	☐	☐
30 mins or more	☐	☐

D. YOUR CHILD'S HOBBIES AND ACTIVITIES

D.1. On a **normal weekday** during term time, how many hours does your child spend watching **television, videos or DVDs?** Please remember to include time before school as well as time after school. **SOURCE: GUI**

- ☐ None
- ☐ Less than an hour
- ☐ 1 hour to less than 3 hours
- ☐ 3 hours to less than 5 hours
- ☐ 5 hours to less than 7 hours
- ☐ 7 hours or more

D.2. On a **normal weekday** during term time, about how many hours does your child spend **reading** for pleasure [NOT during school hours]? Include time when the child reads to themselves or is read to by someone else. Do not include time spent listening to books on audio tapes, records, cds or a computer. **SOURCE: GUI**

- ☐ None
- ☐ Less than an hour
- ☐ 1 hour to less than 3 hours
- ☐ 3 hours to less than 5 hours
- ☐ 5 hours to less than 7 hours
- ☐ 7 hours or more

D.3. On a **normal weekday** during term time, about how many hours does your child spend using the **computer and non-active game consoles (Playstation, X-box etc).** Please include time before school as well as time after school. DO NOT include time spent using computers in school. **SOURCE: GUI**

- ☐ None
- ☐ Less than an hour
- ☐ 1 hour to less than 3 hours
- ☐ 3 hours to less than 5 hours
- ☐ 5 hours to less than 7 hours
- ☐ 7 hours or more

D.4. On a **normal weekday** during term time, about how many hours does your child spend playing **active games consoles such as Nintendo Wii etc?** Please include time before school as well as time after school. **SOURCE: GUI**

- ☐ None
- ☐ Less than an hour
- ☐ 1 hour to less than 3 hours
- ☐ 3 hours to less than 5 hours
- ☐ 5 hours to less than 7 hours
- ☐ 7 hours or more

D.5. On days when your child is given homework, home much time does he or she spend doing **homework**? **SOURCE: GUI**

- ☐ 0 to 15 minutes
- ☐ 16 to 30 minutes
- ☐ 31 minutes to less than 1 hour
- ☐ 1 to less than 1.5 hours
- ☐ 1.5 to less than 2 hours
- ☐ 2 to less than 3 hours
- ☐ 3 to less than 4 hours
- ☐ 4 hours or more

E. YOUR CHILD'S DIET AND DIETARY HABITS

E.1. What type of milk does your child **typically** consume whilst at home? **(Please Tick One) SOURCE: SLÁN**

- ☐ None
- ☐ Whole/ Full fat
- ☐ Low Fat
- ☐ Skimmed
- ☐ Super/ Fortified
- ☐ Other ____________

E.2. Approximately, how much milk did your child drink in the **last 24 hours?** [This refers to the total amount of all milk full cream and skimmed that was drunk]. A small glass of milk contains approximately 100mls while a large glass contains approximately 250mls. **SOURCE: GUI**

- ☐ Up to ½ pint (approx. 250mls)
- ☐ ½ - 1 pint (approx. 250 - 500mls)
- ☐ 1 - 1 ½ pints (approx. 500 - 1000mls)
- ☐ More than 1 ½ pints (more than 1000mls)

E.3. What type of **spread** does your child **usually** use on bread? **(Please Tick One) SOURCE: SLÁN**

- ☐ Butter or hard margarine (e.g. Kerrygold)
- ☐ A low fat or polyunsaturated spread (e.g. LowLow)
- ☐ A cholesterol lowering spread (e.g. Flora Proactive, Kilkeely Gold Low Cholesterol Spread)
- ☐ None
- ☐ Other: ____________

E.4. Does your child usually have something to eat **before going to school? SOURCE: GUI**

- ☐ Yes
- ☐ No

E.5. Does your child usually have a meal in the **evening** during the week? **SOURCE: GUI**

- ☐ Yes
- ☐ No
- ☐ Sometimes

E.6. If yes, does your child usually **sit** at a table for the evening meal? **SOURCE: GUI**

- ☐ Yes
- ☐ No

E.7. Does your child consume fruit? **SOURCE: DEVELOPED BY CCLaS RESEARCH TEAM**

- ☐ Yes
- ☐ No

E.8. Does your child consume vegetables? **SOURCE: DEVELOPED BY CCLaS RESEARCH TEAM**

☐ Yes ☐ No

E.9. How many cans (330ml) or small bottles (up to 500ml) of **soft drinks** does your child usually have per week?

SOURCE: ALSPAC

☐☐ Bottles **OR** ☐☐ Cans

E.10. How many cans (330ml) or small bottles (up to 500ml) of **energy or sports drinks** does your child usually have per week? **SOURCE: ALSPAC**

☐☐ Bottles **OR** ☐☐ Cans

E.11. Has your child had any of the following supplements in the last 12 months? **(Tick all that apply) SOURCE: ALSPAC**

None ☐ Calcium ☐ Vitamin C ☐ Vitamin D ☐

☐ Iron ☐ Cod liver oil ☐ Multivitamins ☐ Other_______________

E.12. How often has your child taken supplements in the **last 12 months? SOURCE: ALSPAC**

☐ Never

☐ Yes, takes them most days **(Please give full name of supplement)** ☐

☐ Yes, takes them occasionally **(Please give full name of supplement)** ☐

E.13. Is your child on any type of **special diet** e.g. vegetarian, vegan, coeliac etc.? **SOURCE: GUI**

☐ Yes ☐ No

If yes, please specify ☐

E.14. Please tick **one** box for each statement below: **SOURCE: CHILD FEEDING QUESTIONNAIRE**

	**Disagree**	**Slightly Disagree**	**Neutral**	**Slightly Agree**	**Agree**
I have to be sure that my child does not eat too many sweets (candy, ice-cream, cake or pastries)	☐	☐	☐	☐	☐
I have to be sure that my child does not eat too many high fat foods	☐	☐	☐	☐	☐
I have to be sure that my child does not eat too much of his/her favourite foods	☐	☐	☐	☐	☐
I intentionally keep some foods out of my child's reach	☐	☐	☐	☐	☐
I offer sweets (candy, ice cream, cake, pastries) to my child as a reward for good behaviour	☐	☐	☐	☐	☐
I offer my child her favourite foods in exchange for good behaviour	☐	☐	☐	☐	☐
If I did not guide or regulate my child's eating, s/he would eat too many junk foods	☐	☐	☐	☐	☐
If I did not guide or regulate my child's eating, s/he would eat too much of his/her favourite foods	☐	☐	☐	☐	☐

F. CURRENT PARENT/GUARDIAN HEALTH

F.1. In general would you say **your** health is...? **SOURCE: GUI**

☐ Excellent ☐ Very good ☐ Good ☐ Fair ☐ Poor

F.2. What is **your** height without shoes? **SOURCE: GUI**

☐ Feet ☐☐ Inches **OR** ☐☐☐.☐ Centimetres ☐Don't Know

F.3. What is **your** weight without clothes and shoes? **SOURCE: GUI**

☐☐ Stone ☐☐ Lbs **OR** ☐☐☐☐☐ Kilograms ☐Don't Know

F.4. Where applicable, what is your **partner's** height without shoes? **SOURCE: GUI**

☐ Feet ☐☐ Inches **OR** ☐☐☐.☐ Centimetres ☐Don't Know

F.5. Where applicable, what is your **partner's** weight without clothes and shoes? **SOURCE: GUI**

☐☐ Stone ☐☐ Lbs **OR** ☐☐☐☐☐ Kilograms ☐Don't Know

F.6. Do you think that you are? **SOURCE: GUI**

- ☐ Very underweight
- ☐ Moderately underweight
- ☐ Slightly underweight
- ☐ About the right weight
- ☐ Slightly overweight
- ☐ Moderately overweight
- ☐ Very overweight
- ☐ Don't know

F.7.How often do you try to lose weight through dieting? **SOURCE: GUI**

- ☐ Very Often
- ☐ Often
- ☐ Sometimes
- ☐ Rarely
- ☐ Never

F.8. Have you ever been told by a doctor that **you or your partner** have, **or** have had any of the following conditions?
SOURCE: MITCHELSTOWN COHORT STUDY

				If **Yes,** Please Answer	
Heart Disease	☐ Yes	☐ No	☐ Don't Know	☐ Me	☐ Partner
Stroke	☐ Yes	☐ No	☐ Don't Know	☐ Me	☐ Partner
Hypertension/ High Blood Pressure	☐ Yes	☐ No	☐ Don't Know	☐ Me	☐ Partner
Diabetes	☐ Yes	☐ No	☐ Don't Know	☐ Me	☐ Partner
Asthma	☐ Yes	☐ No	☐ Don't Know	☐ Me	☐ Partner
Depression	☐ Yes	☐ No	☐ Don't Know	☐ Me	☐ Partner
Gestational Hypertension *(during pregnancy)*	☐ Yes	☐ No	☐ Don't Know	☐ Me	☐ Partner
Gestational Diabetes *(during pregnancy)*	☐ Yes	☐ No	☐ Don't Know	☐ Me	☐ Partner
Other (Please Specify) ________	☐ Yes	☐ No	☐ Don't Know	☐ Me	☐ Partner

Smoking

F.9. Which statement best describes the rules about **smoking inside** your home? **SOURCE: SLÁN**

- ☐ Smoking is not allowed anywhere inside the house
- ☐ Smoking is allowed in some places or at some times
- ☐ Smoking is allowed everywhere inside the house
- ☐ Don't know

F.10. Do you now smoke every day, some days, or not at all? **SOURCE: SLÁN**

☐ Every day ☐ Some days ☐ Not at all

F.11. Have you yourself smoked at least 100 cigarettes in your entire life? (5 packs = 100 cigarettes) **SOURCE: SLÁN**

☐ Yes ☐ No

Alcohol

F.12. How often do you have a drink containing alcohol? **SOURCE: SLÁN**

- ☐ Never
- ☐ Monthly or less
- ☐ 2 - 4 times a month
- ☐ 2-3 times a week
- ☐ 4 or more times a week

F.13. How many drinks containing alcohol do you have on a typical day when you are drinking? **SOURCE: SLÁN**

Please note that a standard drink is:

- a half pint or a glass of beer, lager or cider
- a single measure of spirits (e.g. whiskey, vodka, gin)
- a single glass of wine, sherry or port
- bottle of alcopops (long neck)

☐

F.14. How often do you have **6 or more** [standard] drinks on **one occasion**? **SOURCE: SLÁN**

- ☐ Everyday
- ☐ 5-6 times a week
- ☐ 2-4 times a week
- ☐ Once a week
- ☐ 1-3 times a month
- ☐ Less often
- ☐ Never

Exercise and Physical Activity

**NOTE:** IF 0 DAYS PER WEEK -ENTER 0 HOURS & 0 MINS - ALL 3 SECTIONS OF EACH Q [DAYS, HOURS & MINS MUST BE FILLED IN

F.15. Think about all the **vigorous activities** that you did in the last 7 days. Vigorous physical activities refer to activities that take hard physical effort and make you **breathe much harder** than normal. Think only about those physical activities that you did for at least 10 minutes at a time. During the **last 7 days**, on how many days did you do vigorous physical activities like heavy lifting, digging, aerobics, or fast bicycling? **SOURCE: I-PAQ**

☐ Days per week

If No vigorous physical activities please skip to question F.18

F.16. How much time did you usually spend doing vigorous physical activities on one of those days? **SOURCE: I-PAQ**

☐☐ Hours per day ☐☐ Minutes per day ☐ Don't Know/Not sure

F.17. Think about all the **moderate activities** that you did in the last 7 days. Moderate activities refer to activities that take moderate physical effort and make you **breathe somewhat harder** than normal. Think only about those physical activities that you did for at least 10 minutes at a time. During the **last 7 days**, on how many days did you do moderate physical activities like carrying light loads, bicycling at a regular pace or doubles tennis? Do not include walking. **SOURCE: I-PAQ**

☐ Days per week

If No moderate physical activities please skip to question F.20

F.18. How much time did you usually spend doing moderate physical activities on one of those days? **SOURCE: I-PAQ**

☐☐ Hours per day ☐☐ Minutes per day ☐ Don't Know/Not sure

F.19. Think about the time you spent **walking** in the last 7 days. This includes at work and at home, walking to travel from place to place, and any other walking that you might do solely for recreation, sport, exercise or leisure. During the **last 7 days**, on how many days did you walk for at least 10 minutes at a time? **SOURCE: I-PAQ**

☐ Days per week

If No walking please skip to question F.22

F.20. How much time did you spend walking on one of those days? **SOURCE: I-PAQ**

☐☐ Hours per day ☐☐ Minutes per day ☐ Don't Know/ Not sure

F.21. Think about the time spent **sitting** in the last 7 days. Include time spent in work, at home, while doing course work and during leisure time. This may include time spent sitting at a desk, visiting friends, reading or sitting or lying down to watch television. During the **last 7 days**, how much time did you spend sitting on **a weekday**? **SOURCE: I-PAQ**

☐☐ Hours per day ☐☐ Minutes per day ☐ Don't Know/ Not sure

Well being SOURCE: WEMWBS

STATEMENTS	None of the time	Rarely	Some of the time	Often	All of the time
I've been feeling optimistic about the future	☐	☐	☐	☐	☐
I've been feeling useful	☐	☐	☐	☐	☐
I've been feeling relaxed	☐	☐	☐	☐	☐
I've been feeling interested in other people	☐	☐	☐	☐	☐
I've had energy to spare	☐	☐	☐	☐	☐
I've been dealing with problems well	☐	☐	☐	☐	☐
I've been thinking clearly	☐	☐	☐	☐	☐
I've been feeling good about myself	☐	☐	☐	☐	☐
I've been feeling close to other people	☐	☐	☐	☐	☐
I've been feeling confident	☐	☐	☐	☐	☐
I've been able to make up my own mind about things	☐	☐	☐	☐	☐
I've been feeling loved	☐	☐	☐	☐	☐
I've been interested in new things	☐	☐	☐	☐	☐
I've been feeling cheerful	☐	☐	☐	☐	☐

G. PARENT/GUARDIAN DIET

G.1. How often do you eat fried food? SOURCE: SLÁN

- ☐ Daily
- ☐ 4-6 times a week
- ☐ 1-3 times a week
- ☐ Less than once a week

G.2. How often do you add **salt** to food while **at the table? SOURCE: SLÁN**

- ☐ Always
- ☐ Rarely
- ☐ Usually
- ☐ Never
- ☐ Sometimes

G.3. How often do you add **salt** to food while **cooking? SOURCE: SLÁN**

- ☐ Always
- ☐ Rarely
- ☐ Usually
- ☐ Never
- ☐ Sometimes

G.4. On average, how many portions of fruit do you eat per day? **SOURCE: DEVELOPED BY CCLaS RESEARCH TEAM**

______________ portions per day

G.5. On average, how many portions of vegetables do you eat per day? **SOURCE: DEVELOPED BY CCLaS RESEARCH TEAM**

______________ portions per day

G.6. Did you eat snacks between your meals yesterday? **SOURCE: SLÁN**

- ☐ Yes
- ☐ No

G.6.i. If yes, how many snacks did you eat yesterday: ☐ **SOURCE: SLÁN**

G.6.ii. If yes, what type of snacks did you eat yesterday? **(Please tick all that apply) SOURCE: SLÁN**

- ☐ Biscuits/ Cake
- ☐ Scone
- ☐ Dried fruit
- ☐ Chocolate
- ☐ Yoghurt
- ☐ Vegetables
- ☐ Crisps/Popcorn/ Pretzels
- ☐ Fruit
- ☐ Nuts
- ☐ Other ____________

H. GENERAL FAMILY EATING QUESTIONS SOURCE: SLÁN

H.1. What type of fat/oil would you usually use for cooking? **(Please Tick One)**

☐ Vegetable Oil ☐ Sunflower Oil ☐ Olive Oil/ Rapeseed oil

☐ Lard or dripping ☐ None ☐ Other________________________________

H.2. How often does your family **order take away** in a typical week? **SOURCE: DEVELOPED BY CCLaS RESEARCH**

☐ Daily ☐ 1-3 times a week ☐ 4- 6 times a week ☐ Less than once a week

H.3. How often does your family **eat out** in a typical week? **SOURCE: DEVELOPED BY CCLaS RESEARCH**

☐ Daily ☐ 1-3 times a week ☐ 4- 6 times a week ☐ Less than once a week

H.4. What type of restaurant does your family typically eat out in? **SOURCE: DEVELOPED BY CCLaS RESEARCH**

☐ Standard restaurant ☐ Café ☐ Fast food restaurant ☐ Other: ☐

H.5. Can you afford to buy enough food for your household? **SOURCE: SLÁN**

☐ Always ☐ Sometimes ☐ Usually

☐ Rarely ☐ Never

H.6. During the past 7 days, how many times did all, or most, of your family living in your house eat a meal together?
SOURCE: EAT-1

☐ Never ☐ 1-2 times ☐ 3-4 times

☐ 5-6 times ☐ 7 times ☐ More than 7 times

I. FAMILY BACKGROUND

I.1 How many people in total (including yourself and all children of all ages) regularly live as members of your household? **SOURCE: GUI**

☐ Persons

I.2. For each member of the household, including yourself, could you tell me their **relationship to the study child?**
SOURCE: GUI

Person	Gender	Date Of Birth	Age at last birthday	**Relationship to STUDY CHILD**
1	☐ Male ☐ Female			
2	☐ Male ☐ Female			
3	☐ Male ☐ Female			
4	☐ Male ☐ Female			
5	☐ Male ☐ Female			
6	☐ Male ☐ Female			
7	☐ Male ☐ Female			

I.3 What is your ethnic background? **SOURCE: IRISH CENSUS**

- ☐ Irish
- ☐ Irish Traveller
- ☐ Any other White background
- ☐ African
- ☐ Any other Black background
- ☐ Chinese
- ☐ Any other Asian background
- ☐ Other, incl. mixed background

Please Specify: ☐

I.4. What is your current marital status? **(Please select one answer)SOURCE: MITCHELSTOWN COHORT STUDY**

- ☐ Single
- ☐ Married
- ☐ Cohabiting
- ☐ Separated
- ☐ Divorced
- ☐ Widowed

1.5. Does your family have the use of a car? (Including vans, minibuses etc) **SOURCE: SLÁN**

- ☐ Yes
- ☐ No

I.6. What is the highest level of education **you** have completed to date? **(Please select one answer) SOURCE: GUI**

- ☐ Primary or less
- ☐ Intermediate/ Junior/ Group Certificate or equivalent
- ☐ Leaving Certificate or equivalent
- ☐ Diploma or Certificate
- ☐ Primary degree
- ☐ Postgraduate/ Higher degree
- ☐ Refusal

I.7. Which of these descriptions BEST describes your usual situation in regard to work? (**Please select one answer)**

- ☐ Employee (incl. Apprenticeship or Community Employment)
- ☐ Self employed outside farming
- ☐ Farmer
- ☐ Student Full-time
- ☐ On state training scheme (FAS, Failte Ireland)
- ☐ Unemployed, actively looking for a job
- ☐ Long term sickness or disability
- ☐ Home duties/ looking after home or family
- ☐ Retired
- ☐ Other (specify) **SOURCE: GUI**

I.8. How many hours do you normally work per week, including any regular overtime work? **SOURCE: GUI**

If you work at more than one job, please include the hours in all jobs. ☐ hours

I.9. What is your occupation in this job? (What do you mainly do in your job?) Please describe as fully as possible.

SOURCE: GUI

I.10. Do you supervise or manage any personnel in your job? **SOURCE: GUI**

☐ Yes ☐ No

I.11. If yes, how many people do you supervise or manage? ____ **SOURCE: GUI**

I.12. If self employed, how many employees (if any) do you have? ☐ ☐ N/A
SOURCE: GUI

I.13. Does anyone other than yourself and/or your spouse/partner provide care to the Study Child on a regular basis for 8 hours or more each week? **SOURCE: GUI**

☐ Yes ☐ No

I.14. If yes, is this form of childcare provided in: **SOURCE: DEVELOPED BY CCLaS RESEARCH**

☐ The child's home
☐ A Relatives home
☐ Home of carer-non relative
☐ Centre (crèche, after school activity)

I.15. Approximately how many days per week does the Study Child spend in this form of childcare? **SOURCE: GUI**

☐ days per week

I.16. Is this form of childcare paid or non-paid?

☐ Paid ☐ Non Paid

<u>The remaining questions are about your partner- where applicable, please fill in this section</u>

1.17. Where applicable, what is the highest level of education that **your partner** has completed to date? **(Please select one answer) SOURCE: GUI**

☐ Primary or less
☐ Intermediate/ Junior/ Group Certificate or equivalent
☐ Leaving Certificate or equivalent
☐ Diploma or Certificate
☐ Primary degree
☐ Postgraduate/ Higher degree
☐ Refusal

I.18. Where applicable, which of these descriptions **BEST** describes **your partners** usual situation in regard to work? **(Please select one answer)** **SOURCE: GUI**

- ☐ Employee (incl. Apprenticeship or Community Employment)
- ☐ Self employed outside farming
- ☐ Farmer
- ☐ Student Full-time
- ☐ On state training scheme (FAS, Failte Ireland)
- ☐ Unemployed, actively looking for a job
- ☐ Long term sickness or disability
- ☐ Home duties/ looking after home or family
- ☐ Retired
- ☐ Other (specify)

I.19. How many hours does your partner normally work per week, including any regular overtime work? If your partner works at more than one job, please include the hours in all jobs ☐ Hours **SOURCE: GUI**

I.20. What is your partner's occupation in this job? (What do you mainly do in your job?) Please describe as fully as possible. **SOURCE: GUI**

☐

I.21. Does your partner supervise or manage any personnel in his/her job? **SOURCE: GUI**

☐ Yes ☐ No

I.21. If yes, how many people does he/she supervise or manage? ☐ person/people **SOURCE: GUI**

I.22. If your partner is self employed, how many employees (if any) does he/she have? ☐ employees

☐ N/A **SOURCE: GUI**

Thank you once again for your participation
