# Supplementary material for: Diet, Physical Activity, Lifestyle Behaviors, and Prevalence of Childhood Obesity in Irish Children: The Cork Children’s Lifestyle Study Protocol
Source: JMIR Res Protoc. 2014 Aug 19;3(3):e44. doi: 10.2196/resprot.3140 (PMC4147704; doi:10.2196/resprot.3140)
Supplement: Supplementary file 4 [file resprot_v3i3e44_app4.pdf]

CORK CHILDREN'S LIFESTYLE STUDY

3 DAY FOOD DIARY AND INSTRUCTIONS

START DATE: _______________

FINISH DATE: _______________

PLEASE AFFIX

LABEL HERE

<u>HOW TO FILL IN YOUR FOOD DIARY</u>

We would like to know everything that you eat and drink over the **<u>next 3 days</u>.**

- Eat and drink as you **USUALLY** do.
- Bring your food diary with you **EVERYWHERE** you go. Fill in the food diary at **SCHOOL** and at **HOME.**
- There are **2 PAGES FOR EACH DAY.**
- Use a **NEW LINE** for every food and drink that you eat.
- Write down **EVERYTHING** you eat and drink, **HOW MUCH** of it you had (you can use the pictures to help you with this) and the **TIME** you ate or drank it at.
- List foods such as sandwiches as **SEPERATE** food items. For example, a ham sandwich is written as: 2 slices of white bread, butter and 1 slice of ham.
- Don't forget all those **LITTLE EXTRAS** that you eat with your meals. Some examples are salt, sugar, butter, ketchup and gravy.
- Don't forget to include all **SNACKS AND DRINKS** that you have in between meals. Some examples are biscuits, crisps, fruit and drinks such as tea, water, fizzy drinks and diluted drinks.

- For any **FAST FOOD** or takeaways, write down the **NAME** of the restaurant such as McDonald's or Burger King. You can write this in the **"where"** box.

- For all cooked foods, tell us **HOW** it was **COOKED**. Some ways of cooking foods are frying, boiling, roasting or barbequing.

- Include all **SUPPLEMENTS** you take such as Vitamin C or Cod Liver Oil etc.

USING THE PICTURES - WHICH ONE DID YOU HAVE?

When you are filling out your food diary:

- Use the pictures on pages 3 to 7 to help you decide **how much** of each food you had.
- Use the number written next each picture to fill in the **'how much did you eat or drink'** section of your food diary.
- You can also use the pictures for similar foods to those in the pictures. You can use the '**bread and jam'** pictures for butter or chocolate spread too.
- We have a book with lot of other pictures of foods and drinks. We will show you some more of these pictures in the classroom if you need some extra help.

HERE ARE SOME TIPS TO ESTIMATE PORTION SIZE:

Amount of food	Portion size
**Meat** = the size of a deck of card or the palm of your hand	3 oz
**Cheese** = the of your thumb or a small matchbox	1 oz
**Rice, cereal or pasta** = size of your fist	1 cup
**Rice, cereal or pasta** = small handful or a light bulb	½ cup
A piece of **fruit** = tennis ball	Medium sized
**Butter, nutella or mayonnaise** = thumb tip	1 teaspoon

Figure 1: Yogurt

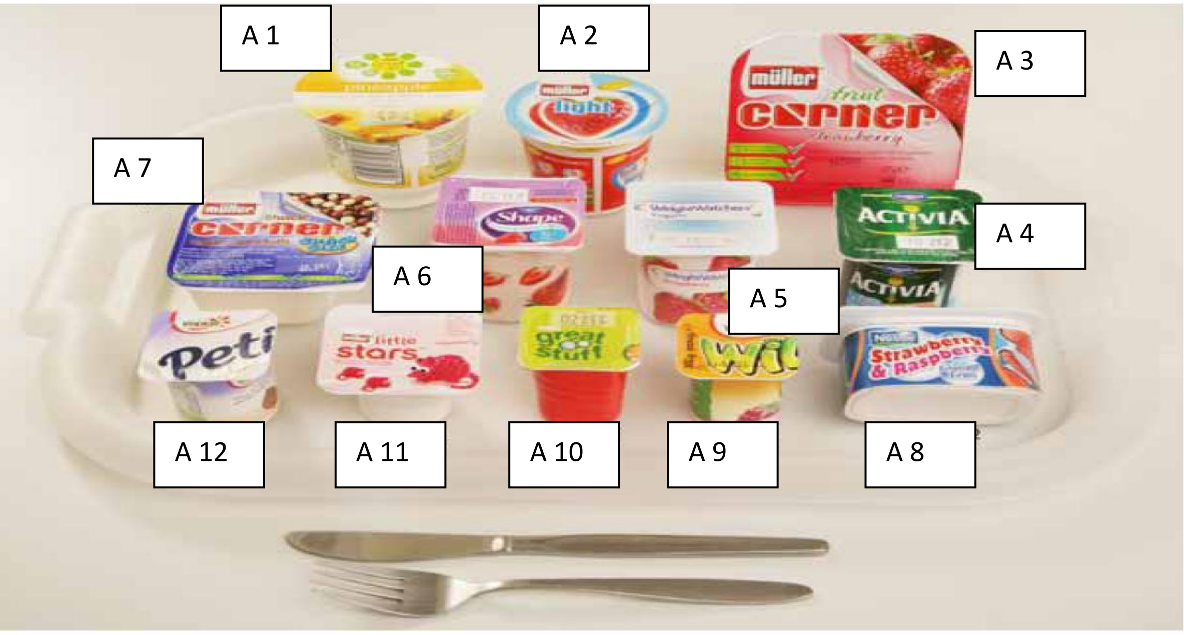

Figure 2: Drinks

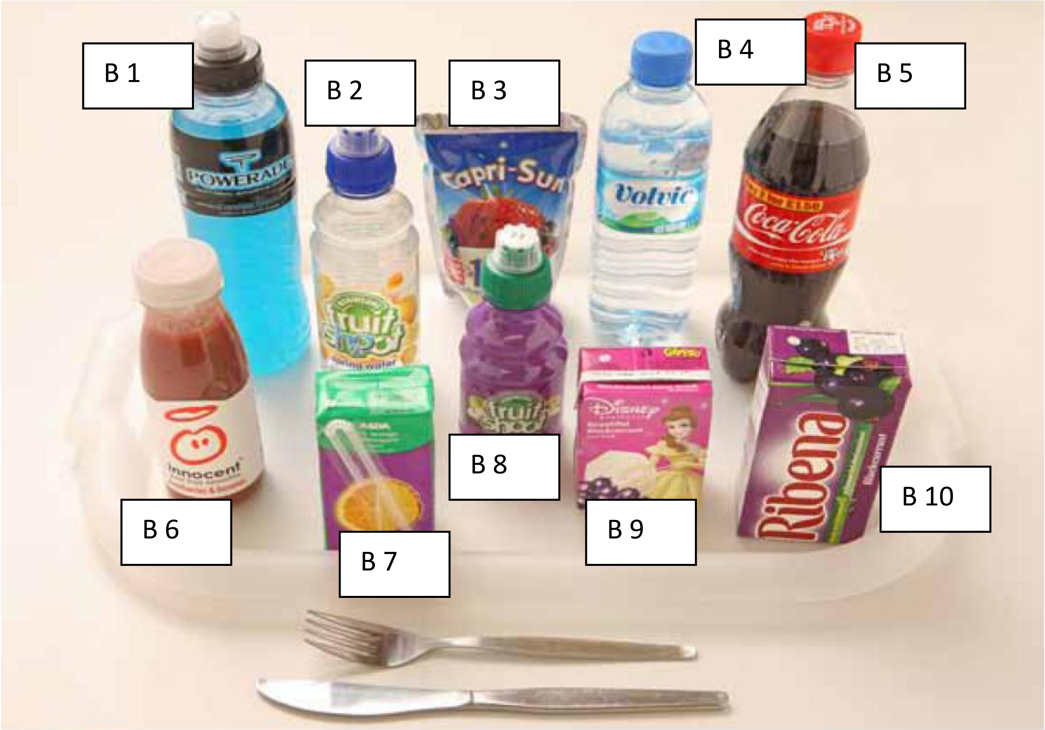

Figure 3: Glass Size

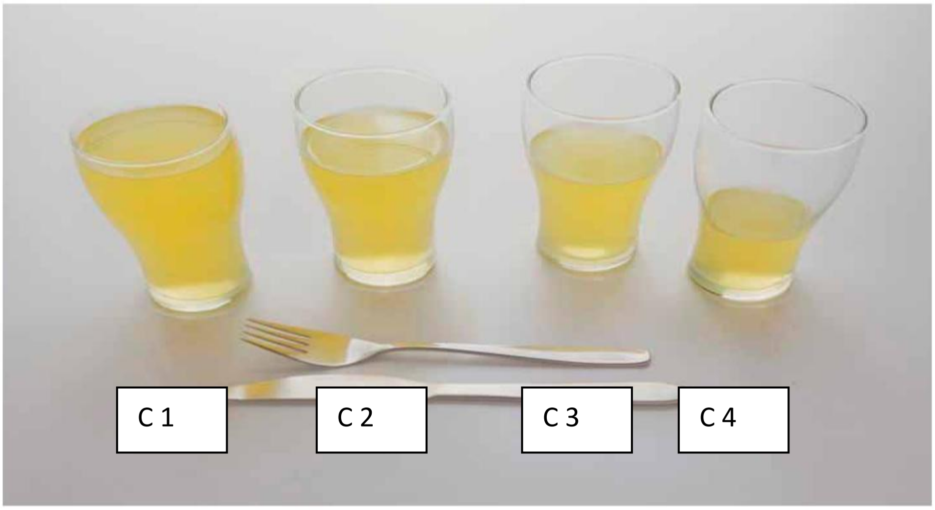

Figure 4: Cereal

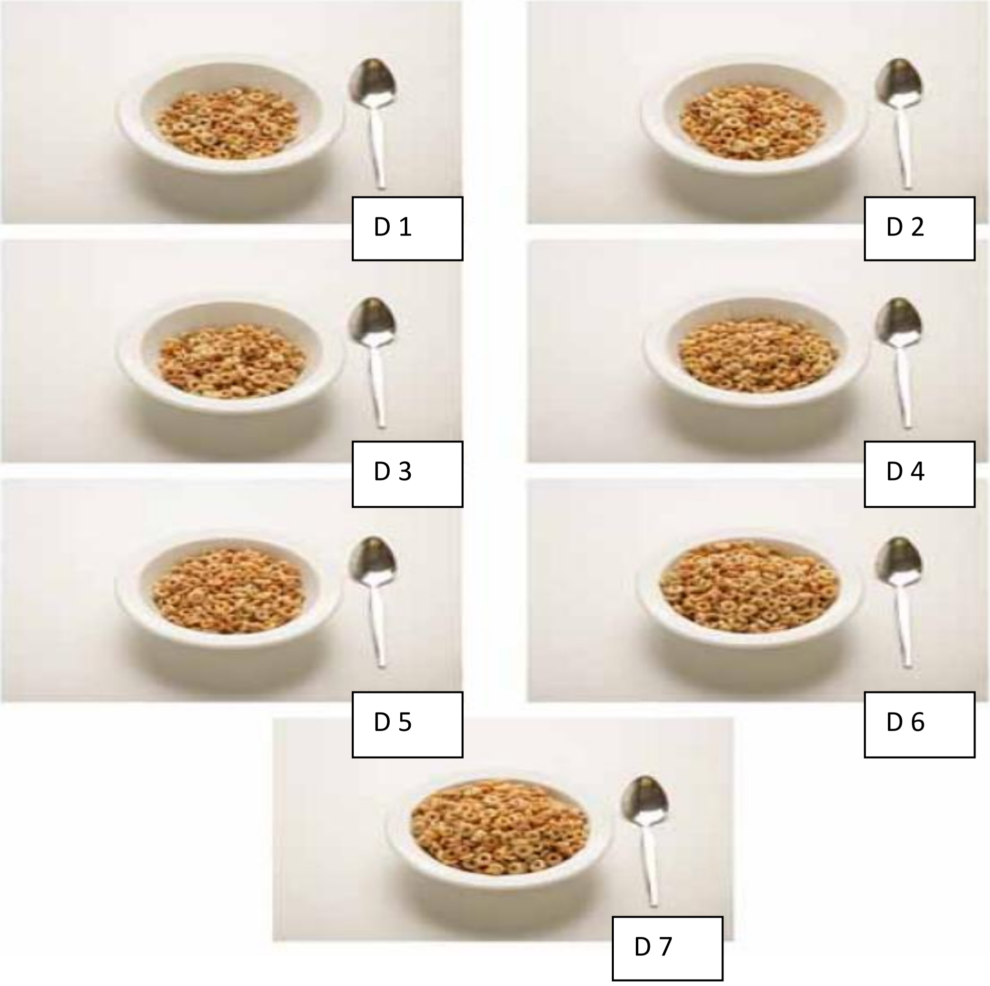

Figure 5: Cereal with milk

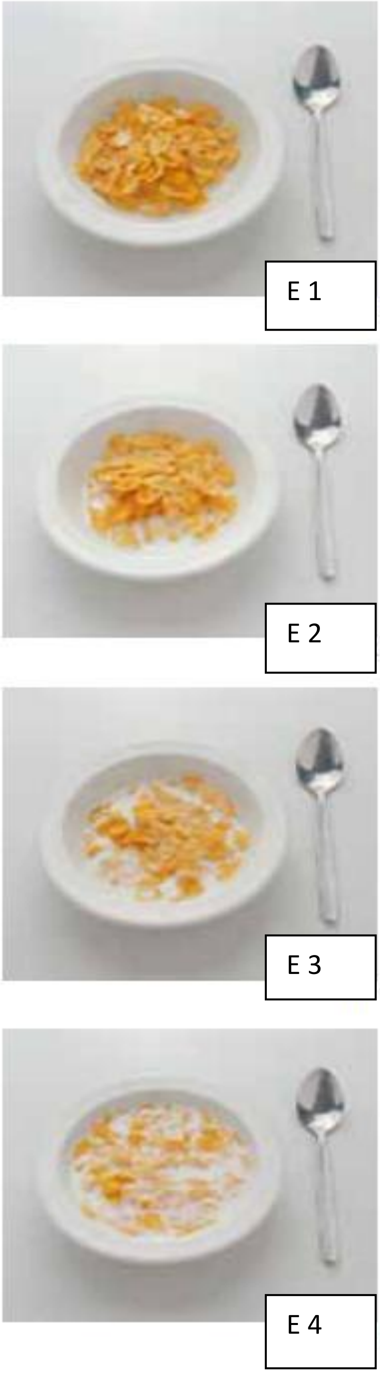

Figure 6: Bread and jam

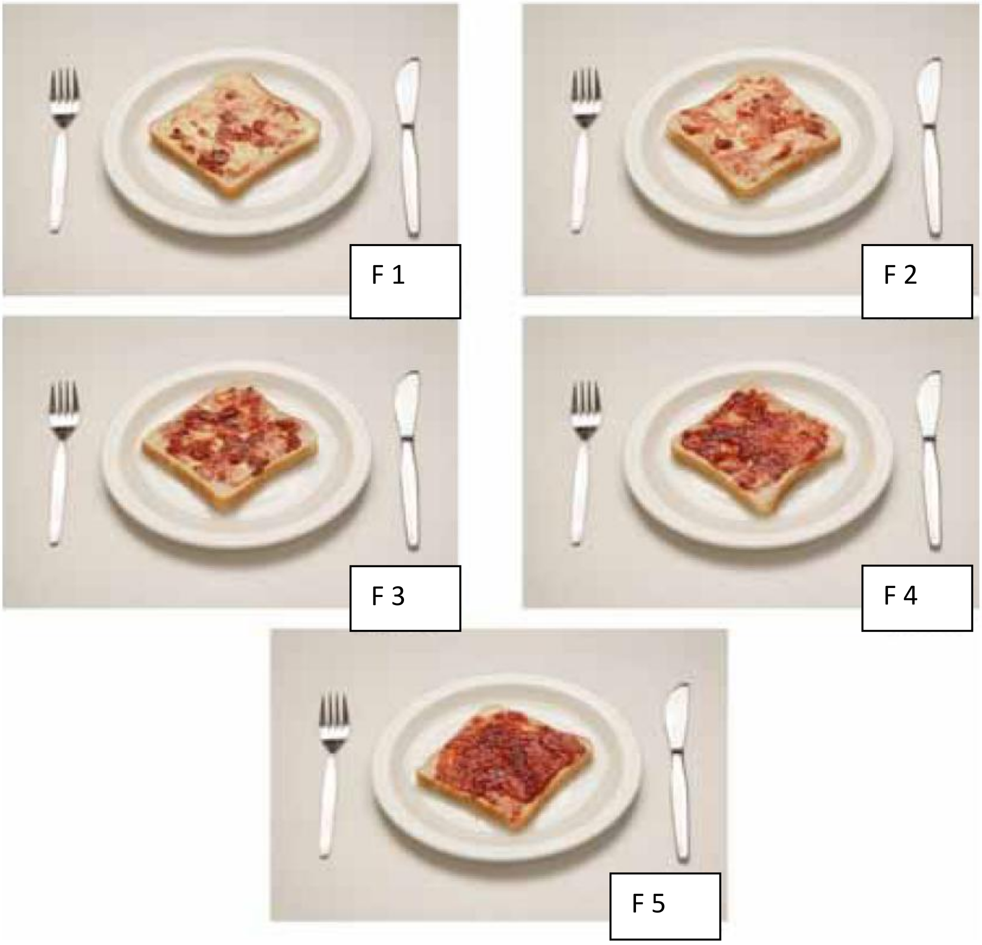

Figure 7: Bread Rolls

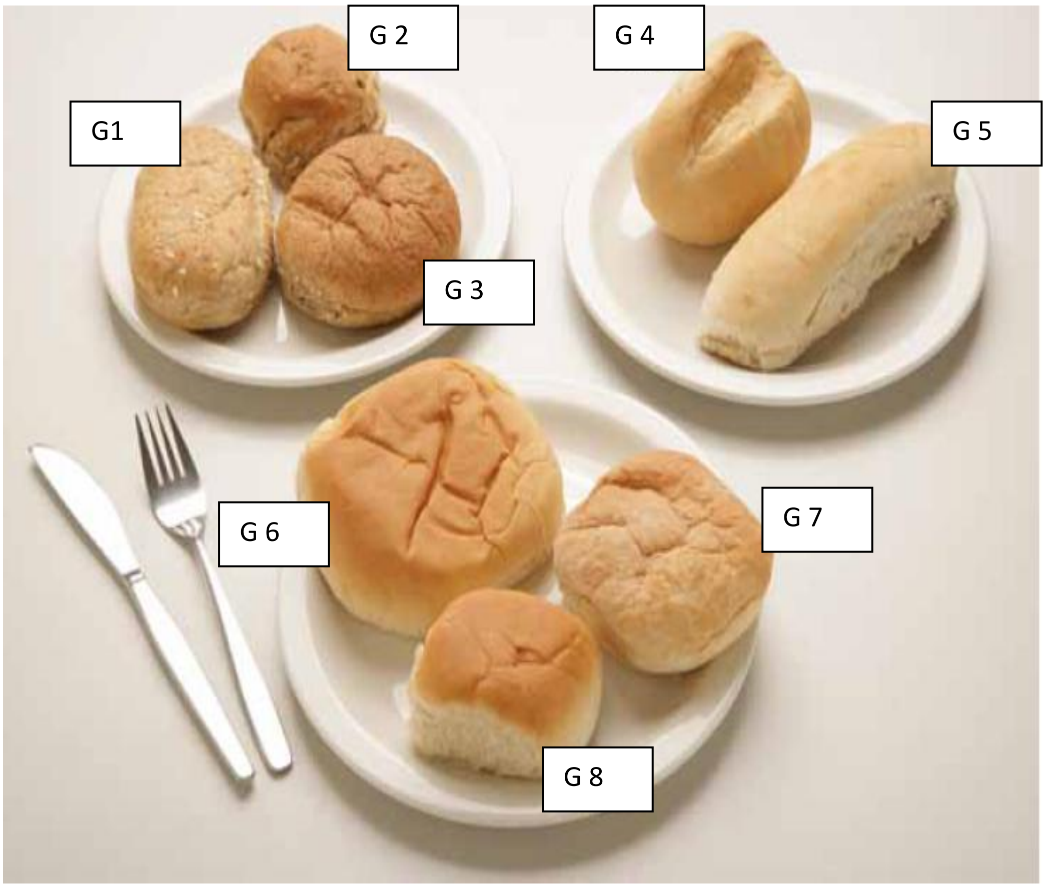

Figure 8: Beef Slices

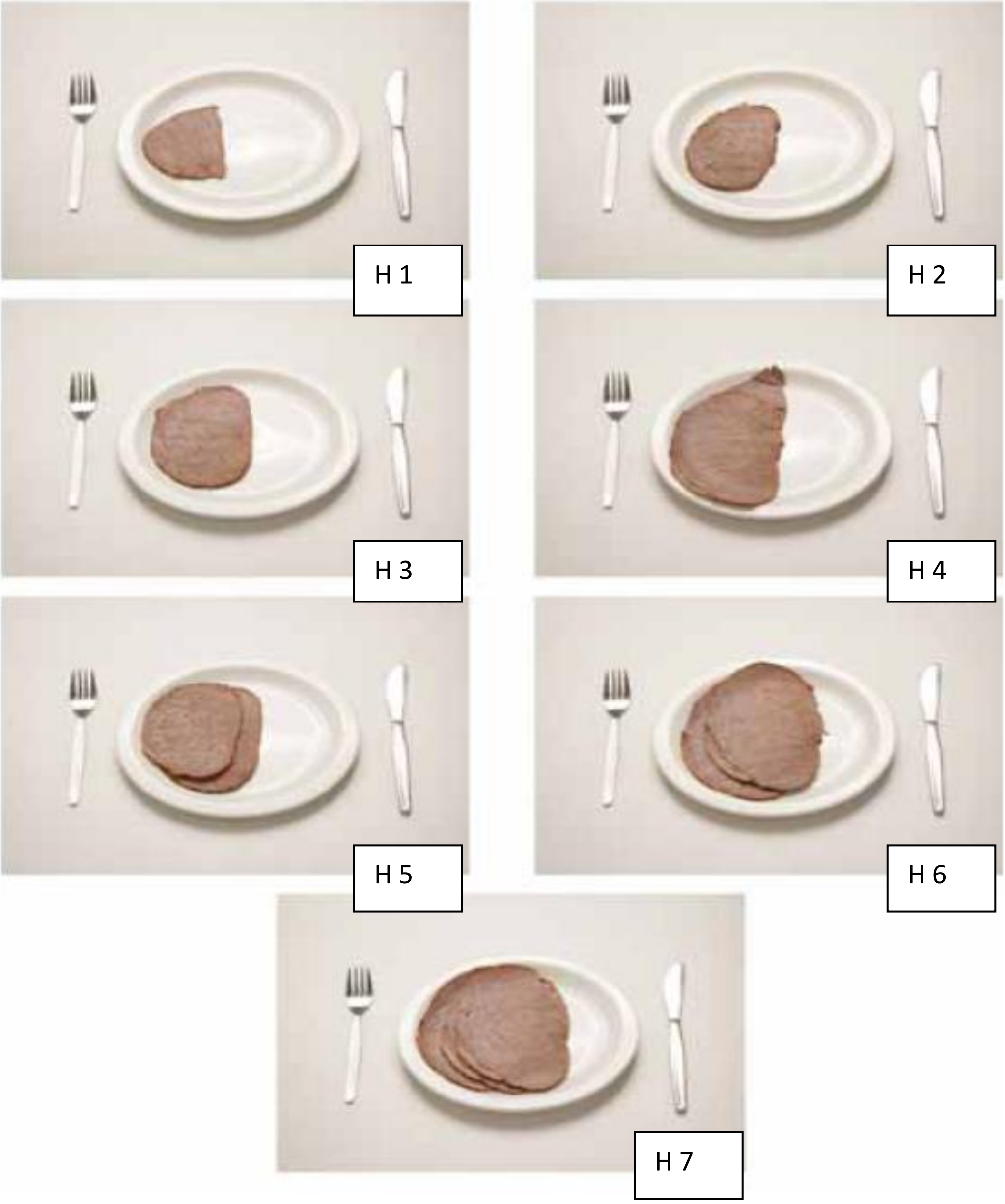

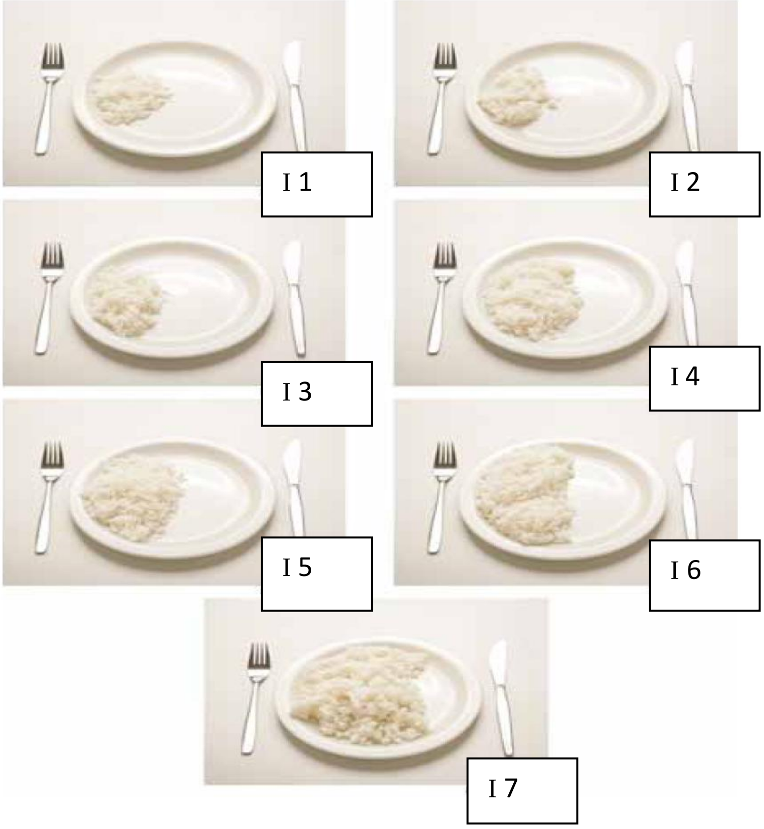

Figure 9: Rice

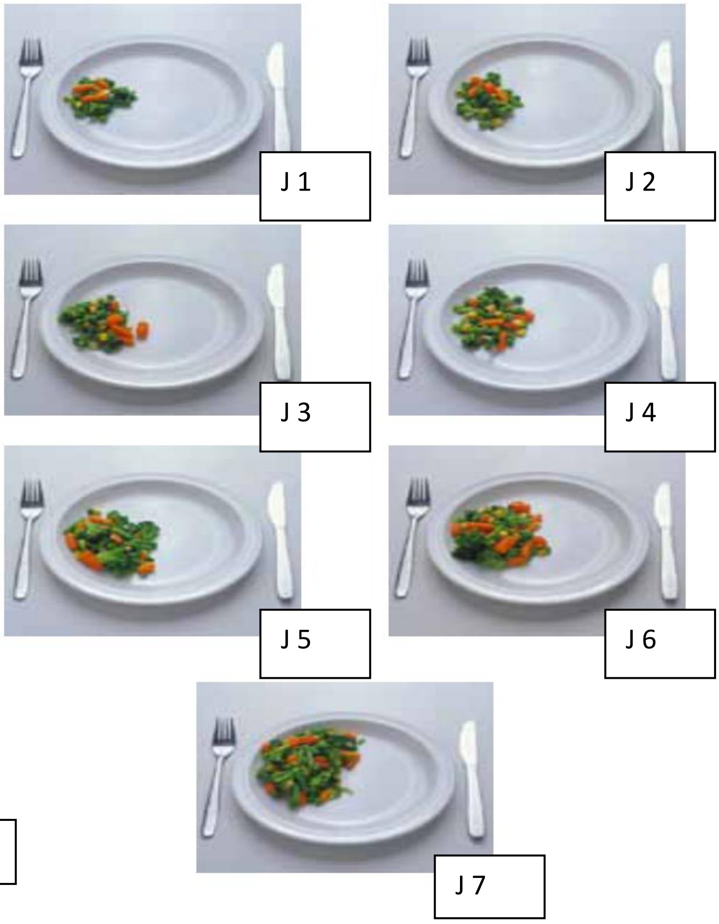

Figure 10: Mixed Vegetables

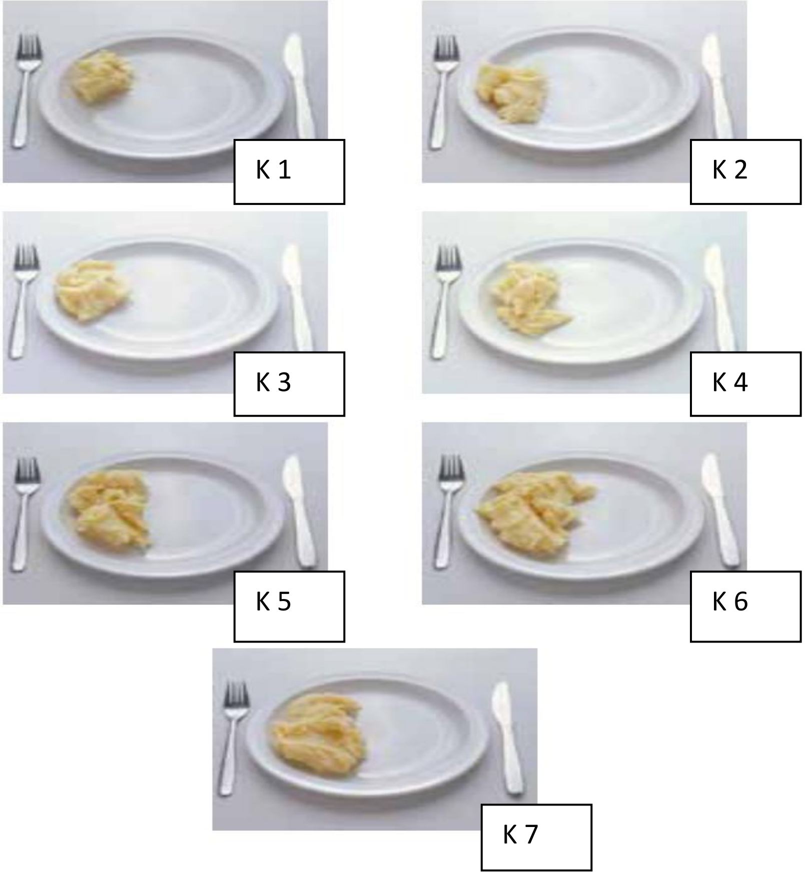

Figure 11: Mashed potato

Figure 12: Pasta

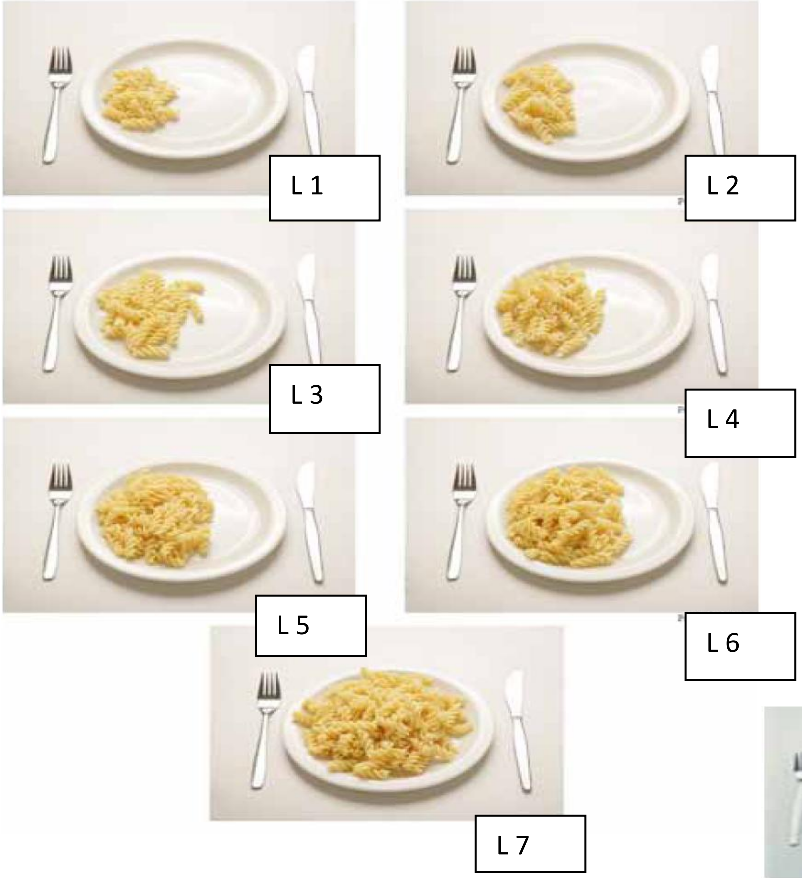

Figure 13: Chips

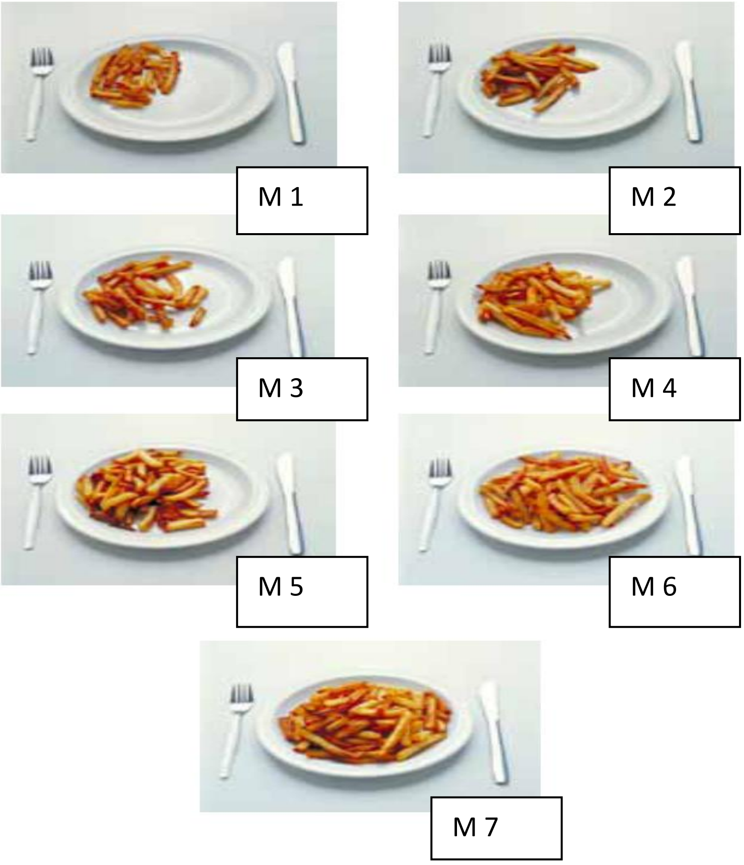

Figure 14: Cook in Sauce

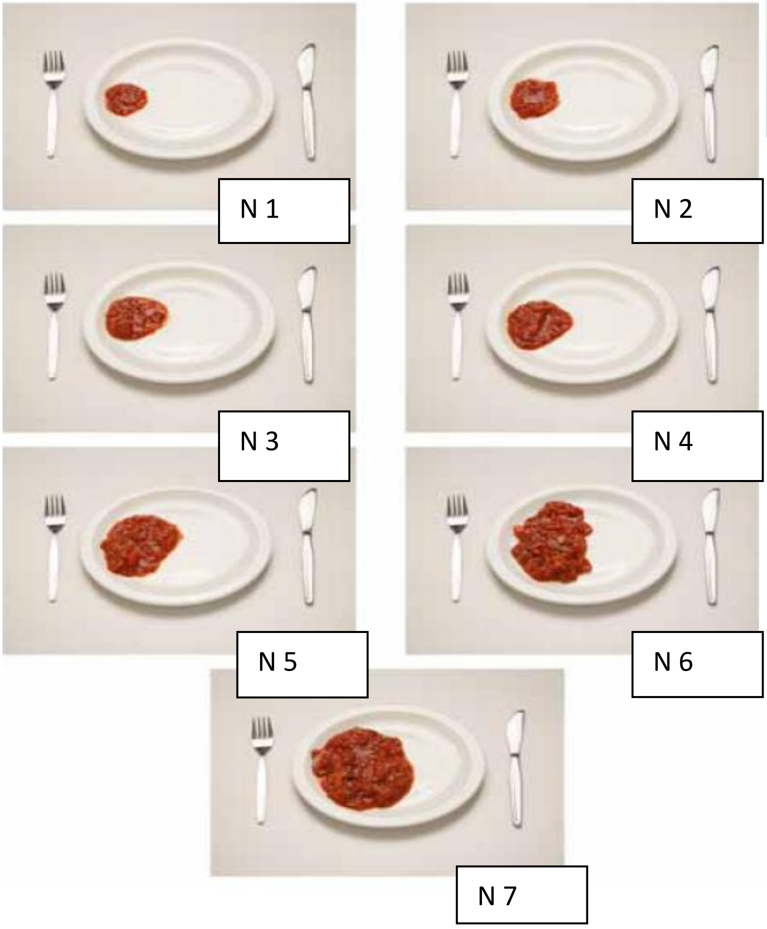

DAY 1: ________________ **DATE:** ________________

What type of FOOD or DRINK did you have?	How much did you EAT or DRINK?	How much did you have LEFTOVER?	If it was cooked, HOW was it COOKED?
**BREAKFAST** Time: ☐☐ : ☐☐ am	Where: ________________		
**MORNING SNACK** Time: ☐☐ : ☐☐ am	Where: ________________		
**LUNCH** Time: ☐☐ : ☐☐ pm	Where: ________________		

What type of FOOD or DRINK did you have?	How much did you EAT or DRINK?	How much did you have LEFTOVER?	If it was cooked, HOW was it COOKED?
**AFTERNOON SNACK** Time: ☐☐ : ☐☐ pm Where: ________________			
**DINNER** Time: ☐☐ : ☐☐ pm Where was this meal prepared: ________________			
**EVENING SNACK** Time: ☐☐ : ☐☐ pm Where: ________________			

DAY 2: ______________ **DATE:** ______________

What type of FOOD or DRINK did you have?	How much did you EAT or DRINK?	How much did you have LEFTOVER?	If it was cooked, HOW was it COOKED?
**BREAKFAST** Time: ☐☐ : ☐☐ am		Where: ______________	
**MORNING SNACK** Time: ☐☐ : ☐☐ am		Where: ______________	
**LUNCH** Time: ☐☐ : ☐☐ pm		Where: ______________	

What type of FOOD or DRINK did you have?	How much did you EAT or DRINK?	How much did you have LEFTOVER?	If it was cooked, HOW was it COOKED?
**AFTERNOON SNACK** Time: __ __ : __ __ pm Where: ______________			
**DINNER** Time: __ __ : __ __ pm Where was this meal prepared: ______________			
**EVENING SNACK** Time: __ __ : __ __ pm Where: ______________			

DAY 3: ________________ **DATE:** ________________

What type of FOOD or DRINK did you have?	How much did you EAT or DRINK?	How much did you have LEFTOVER?	If it was cooked, HOW was it COOKED?
**BREAKFAST** Time: ☐☐ : ☐☐ am		Where: ________________	
**MORNING SNACK** Time: ☐☐ : ☐☐ am		Where: ________________	
**LUNCH** Time: ☐☐ : ☐☐ pm		Where: ________________	

What type of FOOD or DRINK did you have?	How much did you EAT or DRINK?	How much did you have LEFTOVER?	If it was cooked, HOW was it COOKED?
**AFTERNOON SNACK** Time: ☐☐:☐☐ pm	Where: ____________		
**DINNER** Time: ☐☐:☐☐ pm	Where was this meal prepared: ____________		
**EVENING SNACK** Time: ☐☐:☐☐ pm	Where: ____________		
