# Supplementary material for: Diet, Physical Activity, Lifestyle Behaviors, and Prevalence of Childhood Obesity in Irish Children: The Cork Children’s Lifestyle Study Protocol
Source: JMIR Res Protoc. 2014 Aug 19;3(3):e44. doi: 10.2196/resprot.3140 (PMC4147704; doi:10.2196/resprot.3140)
Supplement: Supplementary file 5 [file resprot_v3i3e44_app5.pdf]

Individual, family and environmental factors measured by the CCLaS questionnaires.

Factors		Child questionnaire	Parent/guardian questionnaire	Principal questionnaire
**Individual**				
	Sociodemographic	Gender and age	Gender and age of parent respondent and relationship of parent respondent to study child	Principal gender, school gender mix, school size, and school disadvantaged status
	Birth factors		Birth weight, gestational age, mode of delivery, and breastfeeding	
	Diet	Breakfast consumption, salt use at table, favourite snack and drink, and frequency of consumption of favourite snack and drink	Type and quantity of milk consumed, type of spread typically used, consumption of breakfast, evening meals, fruit and vegetables, quantity of intake of soft drinks and sports drinks, supplement use, special dietary requirements; and parental beliefs, attitudes and practices to child feeding	
	Physical activity	Types and frequency of activities including physical activity during and outside school hours	Frequency of light and hard activity, and mode of transport to and from school	
	Sedentary behaviours	Frequency of use of computer games, games consoles, television, and time spent at homework	Amount of time spent watching television, reading, playing computer games, games consoles, and doing homework	
	Health/ lifestyle	Perception current health and weight status, favourite	Current health status, description of ongoing health issues, perception	

		hobby, and pet ownership	of child weight, and child sleeping patterns	
**Family**				
	Sociodemographic/ family environment	Siblings	Parent reported variables on self and family*: Number of residents in family home, age and relationship of each resident to study child, ethnicity, marital status of parent respondent, car ownership, childcare arrangements, parent education and occupation, and partners education and occupation	
	Parental factors		Parent reported variables on self*: Frequency of consumption of fried foods, fruits, vegetables and salt, snacking patterns, frequency and amount of physical activity, perceived current health status and types of health conditions, perception of current weight status, dieting frequency, self reported height and weight (and of partner where applicable), current smoking, alcohol use, and wellbeing status	
	Family food and eating environment		Parent reported variables on	

			family[*]: Frequency and type of eating out, frequency of ordering takeaway food, frequency of eating family meals together, and affordability of food	
**Environmental**				
		Playground located in neighbourhood, safe play areas in neighbourhood , garden present at family home, and perceived safety of neighbourhood		Provision of food and nutrition education, school health policy available, involvement in and types of health promotion activities, access to and availability of healthy/unhealthy foods in school, provision of school breakfasts and/or lunches, involvement in and types of school sports teams and after school activities, and parent involvement in school

[*] Parent/guardian reported data on child unless specified otherwise
